# Gut Microbiota Fermentation of Digested Almond–Psyllium–Flax Seed-Based Artisan Bread Promotes Mediterranean Diet-Resembling Microbial Community

**DOI:** 10.3390/microorganisms12061189

**Published:** 2024-06-12

**Authors:** Kourtney L. Sprague, Sumudu Rajakaruna, Brant Bandow, Natalie Burchat, Michael Bottomley, Harini Sampath, Oleg Paliy

**Affiliations:** 1 Department of Biochemistry and Molecular Biology, Boonshoft School of Medicine, Wright State University, Dayton, OH 45435, USA; 2 New Jersey Institute for Food, Nutrition, and Health, Rutgers University, New Brunswick, NJ 08901, USA; 3 Statistical Consulting Center, Wright State University, Dayton, OH 45435, USA; 4 Department of Nutritional Sciences, Rutgers University, New Brunswick, NJ 08901, USA

**Keywords:** bread, gut microbiota, short-chain fatty acids, antioxidants, human microbiome

## Abstract

Different modifications of the standard bread recipe have been proposed to improve its nutritional and health benefits. Here, we utilized the *in vitro* Human Gut Simulator (HGS) to assess the fermentation of one such artisan bread by human gut microbiota. Dried and milled bread, composed of almond flour, psyllium husks, and flax seeds as its three main ingredients, was first subjected to an *in vitro* protocol designed to mimic human oro-gastro-intestinal digestion. The bread digest was then supplied to complex human gut microbial communities, replacing the typical Western diet-based medium (WM) of the GHS system. Switching the medium from WM to bread digest resulted in statistically significant alterations in the community structure, encoded functions, produced short-chain fatty acids, and available antioxidants. The abundances of dietary fiber degraders *Enterocloster*, *Mitsuokella*, and *Prevotella* increased; levels of *Gemmiger*, *Faecalibacterium*, and *Blautia* decreased. These community alterations resembled the previously revealed differences in the distal gut microbiota of healthy human subjects consuming typical Mediterranean vs. Western-pattern diets. Therefore, the consumption of bread high in dietary fiber and unsaturated fatty acids might recapitulate the beneficial effects of the Mediterranean diet on the gut microbiota.

## 1. Introduction

The gut microbiome is now recognized as a vital component of the human organism and is involved in many essential functions. Human-associated microbiota develops and evolves together with the host [1], and it participates in numerous biological functions throughout our life, including the digestion of dietary components, prevention of gut pathogen colonization of epithelial surfaces, production of metabolites for various biological processes, the release of beneficial food-bound precursors, reduction of the effects of carcinogens, and even affecting our mood by modulating the human gut–brain axis [2,3,4].

There exists a growing interest in optimizing our diet to not only directly affect the absorption of nutrients but also to support and promote the growth of beneficial members of human gut microbiota [5]. Some studies focused on elucidating which microbes are stimulated by specific foods or dietary components [6,7,8], others assessed the fermentation of the inherently indigestible components in the diet [9,10], and yet another group of reports explored the incorporation of known beneficial dietary components termed prebiotics into different foods [11,12,13].

Among the different foods, bread is a staple food that has been consumed regularly throughout the world for at least the last 14,000 years of human history [14]. Made of various starch-containing species of domesticated cereal grasses such as wheat, it also incorporates other products such as sugar, salt, and vinegar to improve its taste, texture, look, and palatability. In industrialized countries, a substantial portion of bread products is made from bleached and refined wheat flour. During the refinement process, the majority of dietary fiber found in the bran and germ layers is removed from the grains, leaving the flour highly enriched in starch, which is easily digested by intestinal enzymes, and in the gluten proteins, glutenin and gliadin [15]. Refined wheat flour products have a high glycemic index [16], can aggravate celiac disease due to the presence of gluten [17], and have been associated with metabolic disease [15].

Owing to the recognized nutritional and health limitations of standard bread products, different alternative formulations have been developed and tried [18,19,20]. These usually substitute or supplement refined wheat flour with other ingredients in hopes of making such bread products healthier. Here, we studied the oro-gastro-intestinal (OGI) digestion and microbial fermentation of one of such artisan breads manufactured by Uprising Foods, Inc. (abbreviated “UF bread”). The bread was based on the almond flour and also contained substantial amounts of psyllium husks and flax seeds. Almonds and their derivatives have recently gained interest as prebiotic additions to foods due to being good sources of antioxidants, in addition to their prebiotic properties [20,21]. Almond consumption has also been observed to increase beneficial short-chain fatty acid (SCFA) production in humans [22]. On the other hand, dietary incorporation of psyllium husks has long been observed to alleviate constipation symptoms, in addition to promoting beneficial members of the gut communities [23]. For instance, the consumption of psyllium promoted *Veillonella* genus members while decreasing the members of *Subdoligranulum* in a randomized controlled trial in healthy subjects, whereas more prominent shifts, including the increased abundances of butyrate-producing bacterial genera *Lachnospira* and *Faecalibacterium*, were observed for patients with constipation [24]. Finally, flax seeds have long interested researchers due to their high concentration of lignans, which are converted to enterolignans by human gut microbiota and exert health benefits [25]. For instance, Lagkouvardos et al. [26] found that the consumption of flax seeds for six weeks resulted in a significant increase in the blood concentration of enterolignans, underscoring a possible role of members of the family Ruminococcaceae in the regulation of enterolignan production. Flax seeds are also a rich source of omega-3 fatty acids, particularly alpha-linolenic acid, whose consumption is associated with reduced inflammation and improved metabolic health [27].

Our current knowledge of such dietary incorporations is primarily based on the effect of a single compound at a time. Hence, evaluations of commercial products with beneficial dietary constituents are warranted to better understand the combined effects of these compounds. Here, hypothesizing that fiber-rich bread formulation can better support the growth of beneficial human gut microbes in comparison with a Western diet-mimicking medium, we evaluated the digestion, availability, and fermentation of the artisan UF bread by the human gut microbiota in the *in vitro* Human Gut Simulator system [28].

## 2. Materials and Methods

### 2.1. Preparation and In Vitro Digestion of Bread

Fresh bread loaves were obtained from Uprising Foods, Inc. Nutritional composition was listed as 160 calories, 11 g total fats, 11 g total carbohydrates, including 9 g of dietary fiber, and 6 g of proteins per one slice (72 g). Bread was cut into small cubes approximately 1–1.5 cm per side, and these were subjected to prolonged drying at 50 °C to ensure the removal of moisture with minimal chemical changes. Drying times and temperatures were derived from multiple trials. Maximum moisture removal was assured through repeated weight measurements in hourly intervals till no reduction in weight was observed. Dried bread was milled on a ZM200 ultra centrifugal mill (Retsch GmbH, Haan, Germany).

**Figure 1:**
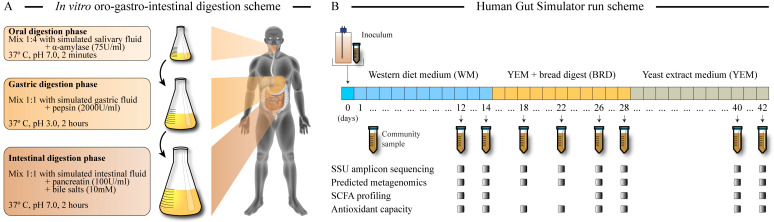
**Experimental design.** Panel (**A**) depicts the steps of the *in vitro* oro-gastro-intestinal digestion of UF bread. Panel (**B**) shows the longitudinal design of the *in vitro* Human Gut Simulator runs. Sample collection time points are indicated by a sample tube, and the analyses that were performed on each collected sample are tabulated. The medium was switched from a standard Western diet medium (WM) to the bread digest mixed with yeast extract and salts (YEM + bread) after taking samples on day 14 and then again to the yeast extract medium (YEM) on day 28.

Milled bread was subjected to an *in vitro* digestion protocol designed to mimic the human OGI digestion of foods. We used a previously described approach [10] with slight modifications. Briefly, *in vitro* digestion consisted of three phases, as summarized in Figure 1A. For enzymatic digestions, pepsin was purchased from Sigma-Aldrich (St Louis, MO, USA), and the enzymatic activity assay was performed as described [29]. Amylase and pancreatin were purchased from MP Biomedicals (Irvine, CA, USA) for which the enzymatic assay values were provided by the manufacturer. Salt solutions were made as described in the referenced publication [10] and mimicked luminal contents of saliva (SSF, simulated salivary fluid), stomach (SGF, simulated gastric fluid), and small intestine (SIF, simulated intestinal fluid) [29]. Wherever necessary, pH adjustments were made using 1M HCl or 1M NaOH. Complete digestion procedure was carried out with continuous agitation at a constant 37 °C temperature measured with a thermal probe (Omega Engineering, CT).

The *in vitro* digestion consisted of three phases (see Figure 1A). During the oral phase, milled bread was mixed with SSF containing 75 U/mL of α-amylase and was maintained for 2 min at pH 7. Due to the extreme hygroscopic nature of the bread powder, a 1:4 bread/SSF weight-to-volume ratio was used to ensure proper mixing and digestion. This oral bolus was mixed with equal volume of SGF containing pepsin (2000 U/mL) and was maintained for 2 h at pH 3. This process was equivalent to human gastric digestion. The gastric chyme was then mixed in a 1:1 ratio with SIF containing pancreatin (100 U/mL) and bile solution (10 mM) and maintained for 2 h at pH 7 to mimic human intestinal digestion. The resulting mix was centrifuged in 50 mL aliquots at 6000 rpm, and supernatant, which represented digestible and absorbable fraction, and pellet (undigested residue) were collected separately, homogenized, and stored at −70 °C till use. For comparison, white and whole wheat bread loaves were purchased from a local supermarket (Meijer, OH, USA) and were cut, dried, milled, and then subjected to the same OGI digestion. The percent of digestible and undigested fractions for each bread are listed in Table 1.

### 2.2. In Vitro Human Gut Simulator (HGS) Experiments

Gut microbial fermentation of the digested UF bread was carried out in the *in vitro* HGS system, which had been previously described and validated [28]. HGS is a fully anaerobic system comprised of five linked compartments: a medium reservoir, a proximal colon vessel, a transverse colon vessel, a distal colon vessel, and a waste collector. All compartments are connected through medium transfer tubes and are “powered” by peristaltic pumps. The medium reservoir contains a nutrient medium that was designed to mimic the contents that reach the gut of an adult after digestion of a Western-type meal (called Western diet medium, or WM) [28]. At day ‘0′, each vessel was inoculated with a homogenized fecal inoculum prepared from fresh fecal samples obtained from three healthy adults (two North Americans and one Western European) with no recent antibiotic or dietary supplement use and without any recent gastrointestinal illnesses. Following the inoculation, HGS was operated for six weeks across three phases, during which three different media were provided, as shown in Figure 1B. Throughout the run, the contents in the medium reservoir and vessels were continuously agitated, and vessel pH was maintained at 6.0, 6.5, and 7.0, respectively, in accordance with the observed colonic pH in these regions [30]. The system was sparged twice daily with a mixture of N_2_ and CO_2_ to maintain the oxygen-depleted environment, and resazurin was used to attest to the anaerobicity of each vessel’s environment.

Three replicate runs were performed, with each run comprising three different phases that differed in the composition of the medium provided to the proximal vessel (Figure 1B). The communities were kept in each phase for two weeks based on the previous observation that between 10 and 14 days are necessary to stabilize complex microbiota communities in the HGS system [28]. In phase 1, a modified version of our standard WM was provided for the first two weeks to establish a stable microbiota community in each vessel [10]. The modifications included the use of mono- and diacylglycerols instead of free fatty acids [31] and the use of resistant starch in place of regular, easily digestible starch. At the end of day 14, the medium reservoir was changed to contain the UF bread digest medium, denoted “BRD” or “YEM + bread”. This medium had the same salt composition as WM, whereas all WM organic components (with the exception of yeast extract) were replaced by an equal weight of bread digest pellet (see Table 2). An additional 10% of digest supernatant was also added, which represented digested soluble compounds escaping absorption in the small intestine [6]. The HGS system was maintained on the BRD medium for a further two weeks (Figure 1B), and the medium was switched again at the end of day 28 to the yeast extract medium (YEM) containing salts and yeast extract in concentrations matching both the WM and BRD media. This medium served as a negative control for the BRD medium because it contained identical amounts of all BRD components with the exception of bread residue and digest. This allowed us to obtain proof of bread digest utilization by microbial communities and to distinguish microbiota members that were capable of fermenting digested bread nutrients, along with syntrophs.

**Table 2:** Medium composition, g L^−1^.

*Medium Component*	WM *	YEM + Bread *	YEM *
*Digested UF bread*			
Pellet (dry residue) ^†^	-	27.0	-
Digest supernatant ^‡^	-	34.3	-
*Carbohydrates*			
Arabinogalactan	1.8	-	-
Guar gum	0.9	-	-
Inulin	0.9	-	-
Pectin	1.8	-	-
Starch	4.4	-	-
Xylan	0.9	-	-
Cellobiose	0.9	-	-
Glucose	0.5	-	-
Fructose	0.5	-	-
*Proteins*			
Peptone	3.3	-	-
Casein	2.0	-	-
*Lipids*			
Mono + diacylglycerides	5.4	-	-
*Mucin*	4.0	-	-
*Yeast extract*	3.0	3.0	3.0
*Vitamins*	1.0	1.0	1.0
*Salts, other components*	14.1	14.1	14.1
Bile salts	0.2	0.2 **	0.2
Pancreatin	0.1	0.1 **	0.1

* WM: Western diet medium; YEM + bread: bread digest-based medium; YEM: yeast extract medium; ^†^ Calculated wet weight equivalent: 220 g per liter of the medium; ^‡^ Wet weight: 10% of the total digestion supernatant; ** amounts added during oro-gastro-intestinal digestion.

Samples for microbial and metabolite analyses were drawn from each vessel regularly (Figure 1B) and were stored at −70 °C. Cell densities in each vessel were obtained via phase contrast microscopy with a Spencer hemocytometer.

### 2.3. Microbiota Analysis

Culture samples were centrifuged to separate them into cell pellets, which were used for bacterial genomic DNA isolation using a ZR bacterial DNA isolation kit (Zymo Research, Tustin, USA), and the supernatants, which were used for SCFA and antioxidant measurements. Isolated bacterial genomic DNA was subjected to the 16S rRNA gene amplification of its V4 hypervariable region in a PCR reaction consisting of 4 linear and 25 exponential cycles. This combination of cycles was found previously to reduce the known biases in PCR [10,32].

Gene amplicon samples were purified using AxyPrep magnetic beads (Axygen Scientific, Inc., Union City, NJ, USA), and sequence libraries were prepared with the Ion PGM Hi-Q View OT2 kit (Thermo Fisher Scientific, Waltham, MA, USA) according to manufacturer’s guidelines. High-throughput sequencing was performed on the Ion Torrent Personal Genome Machine using multiple Ion 318 and 316 chips and the Ion PGM Hi-Q View Sequencing kit (Thermo Fisher Scientific). After quality filtering, we obtained an average of 16,447 sequence reads per sample. Obtained reads were processed in QIIME [33] using our default pipeline [5]. Taxon annotation was based on the Ribosomal Database Project Classifier v2.14 and 16S rRNA training set 19. Obtained reads were converted into cell counts via the 16S rRNA gene copy adjustment procedure that we described previously [10]. Cell counts of all samples were multiplied to match the cell density of each sample obtained via phase contrast microscopy [28]. This merged, cell density adjusted dataset of cell counts was used for all downstream analyses. Cumulative table of genus-level cell densities in all samples is provided in Supplementary Table S1.

### 2.4. Short-Chain Fatty Acid Measurements

HGS aliquots were centrifuged at 13,000× *g* for 5 min, and the supernatant was filtered through a 0.22 µm nylon filter. The supernatant samples were acidified with HCl, and the SCFAs were extracted using methyl tert-butyl ether [34]. SCFAs were analyzed on a DB-Wax GC column (Agilent Technologies, Santa Clara, USA) via gas chromatography–mass spectrometry (GC-MS) performed at the Quantitative Lipidomics Facility at Rutgers University. Eight different SCFAs were measured: acetate, propionate, isobutyrate, butyrate, isovalerate, valerate, hexanoate, and heptanoate. Methyl hexanoate (1 mM, Thermo Fisher Scientific) was added as an internal standard, which was used to correct for injection variability between samples and minor changes in the instrument response. Three independent replicate extractions were performed per sample. The GC-MS system consisted of an Agilent 7890A (Agilent Technologies, Santa Clara, CA, USA), equipped with an automatic liquid sampler MPS2 (Gerstel, Mulheim, Germany) and coupled to an Agilent 5975C mass selective detector. The GC was fitted with a high polarity, polyethylene glycol, fused silica capillary column DB-WAXetr (30 m, 0.25 mm ID, 0.25 μm film thickness), and helium was used as the carrier gas at 1 mL/min. Injection was made in a splitless mode with an injection volume of 1 μL and an injector temperature of 250 °C. Data acquisition was performed using the ChemStation software https://www.agilent.com.cn/zh-cn/product/software-informatics/analytical-software-suite/chromatography-data-systems/openlab-cds/make-the-switch/chemstation (Hewlett-Packard, Palo Alto, USA). Identification of the short-chain fatty acids was based on the retention time of standard compounds and with the assistance of the NIST 08 libraries. SCFA absolute concentrations were determined based on standard curves generated for each individual fatty acid.

### 2.5. Antioxidant Capacity Measurements

Antioxidant capacity of samples was estimated using an ABTS assay adapted to a microplate reader (FLUOStar Omega, BMG Labtech Inc., Cary, NC, USA) as conducted previously [9]. In the assay, 2,2′-azino-bis(3-ethylbenzothiazoline-6-sulfonic acid) diammonium salt (ABTS) is oxidized by potassium persulfate to form an ABTS-radical cation, which has a characteristic blue–green color. The assay measures the antioxidant capacity of samples to prevent or revert back this oxidation in comparison with a Trolox standard. The reactions combined 280 μL of the working ABTS solution with 20 μL of a sample or Trolox standard, and the color development was measured spectrophotometrically in triplicates. A Trolox standard curve was constructed by using a range of Trolox concentrations between 0.01 and 0.1 mg mL^−1^, and the obtained results were expressed as mmol (Trolox equivalents) per liter of sample.

### 2.6. Data Analyses

All statistical and multivariate analyses were carried out in R, Python, and Matlab. Multivariate ordination analyses included principal components analysis (PCA), phylogenetic UniFrac distance-based principal coordinates analysis (UF-PCoA), and canonical correspondence analysis (CCA). Statistical significance of group separation in ordination space was based on the permutation of the Davies–Bouldin index, as we described previously [35]. UniFrac distance-based principal response curves (UF-dbPRC) [36] were generated to interrogate temporal changes in microbiota composition upon fermentation of digested bread as performed previously [10]. PICRUSt2 and STAMP algorithms were used to evaluate the predicted encoded functions of profiled microbial communities [37]. Statistical significance of the differential pathway encoding between groups was calculated with Welch’s *t*-test with Benjamini–Hochberg correction for multiple hypothesis testing [38]. Pathway was defined as differentially encoded (DE) if it was at least 2-fold more prominent in either WM or BRD communities at the α ≤ 0.01 significance level. The statistical significance of the differences in measured values among vessels or media was calculated with repeated measures ANOVA (RM ANOVA) unless stated otherwise.

## 3. Results

### 3.1. Oro-Gastro-Intestinal Digestion of Artisan Bread

In this study, we have explored the digestion and gut microbiota fermentation of one of the artisan breads formulated from high-quality ingredients. Produced by Uprising Foods, Inc., (Cincinnati, OH, USA) the tested bread contained almond flour, egg whites, psyllium husks, flax seeds, apple cider vinegar, baking powder, and salt. No other ingredients were added, including no wheat or other cereal products. The dried and milled UF bread, together with standard supermarket white and whole wheat breads, were subjected to an *in vitro* oro-gastro-intestinal digestion schematically shown in Figure 1A. Much less of the UF bread was digested in this process compared with the wheat and white varieties (Table 1): 70.6% of the UF bread remained undigested and thus available to gut microbiota in the large intestine, compared with 20.6% and 23.4% undigested fractions for supermarket’s white and wheat breads, respectively.

### 3.2. Fermentation of Artisan Bread in the Human Gut Simulator

The HGS system was used to carry out a three-phase cultivation of human gut microbiota following a procedure outlined in Figure 1B. Western diet medium represented the food milieu entering the colon in a subject consuming a typical Western diet (relatively high in animal proteins and fats). In the second phase, the medium was switched to the basal yeast extract medium supplemented with digested UF bread. Phase three was a control medium only containing yeast extract, vitamins, and salts (see Table 2). This combination of three phases of HGS operation allowed us to (i) compare community fermentation of nutrients obtained from the Western diet vs. UF bread and (ii) separate the specific effects of UF bread fermentation from those arising due to the lack of Western diet nutrients.

**Figure 2:**
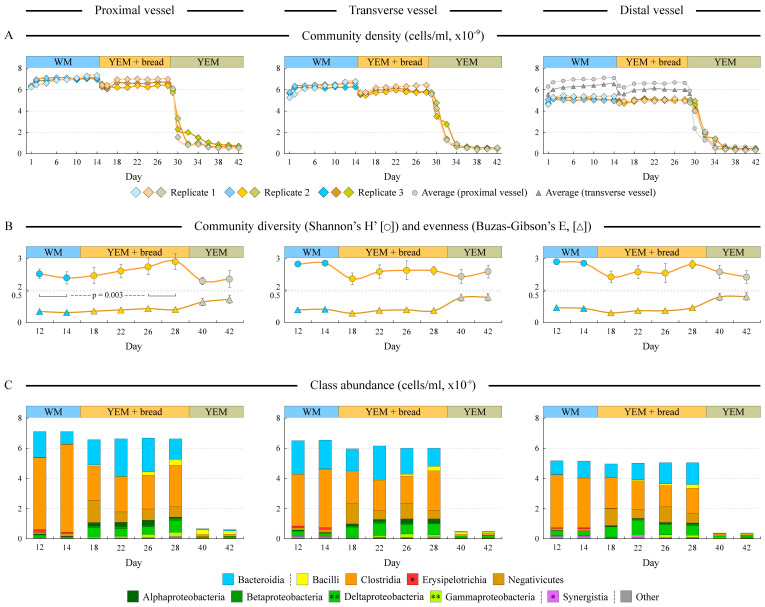
**Dynamic changes in community density and composition.** Different columns represent data for the three consecutively linked vessels simulating proximal, transverse, and distal colon, as shown. Panel (**A**) displays the cell density in each vessel. Panel (**B**) visualizes the calculated average community diversity and evenness in the profiled samples as shown. Error bars represent the standard error of the mean (*n* = 3). Statistical significance between WM and YEM + bread samples (exceeding the α = 0.05 level) was achieved for the diversity values of the proximal vessel community. Panel (**C**) shows the cumulative cell density of different bacterial classes at each time point; each column represents the average class density among three replicate runs. Classes are grouped by their phylum assignment. Stars in class legend denote statistical significance of class abundance differences between WM and YEM + bread samples; **: *p* < 0.01, *: *p* < 0.05 (based on RM ANOVA of the log-transformed abundances).

Three independent runs were carried out, which revealed a good concordance of community densities in each phase of the experiments (see Figure 2A). The bread digest-based medium was designed to provide the same amount of organic compounds per liter of medium as WM. Thus, it is not surprising that we observed only minor differences in the optimal community density reached on WM and BRD (=YEM + bread) media (5.9%, 6.3%, and 2.8% decreased density on BRD vs. WM in proximal, transverse, and distal vessels, respectively), likely explained by the less energy-efficient fermentation of complex polysaccharides in the BRD medium. The medium switch led to a transient drop in the culture density in the proximal and transverse vessels; however, the cell counts recovered within 2–4 days (Figure 2A). In contrast, the YEM medium supported significantly lower community density (less than 10% of that in WM), consistent with the nutrient-poor composition of this growth environment and confirming the use of bread compounds in the YEM + bread phase.

### 3.3. Microbial Composition Differs among Supplied Media

Microbial community diversity and evenness were comparable for the first two phases of HGS experiments in all three simulated regions (Figure 2B). In the proximal vessel, the diversity actually increased upon the switch to the bread digest medium (*p* = 0.003); no statistically significant differences were found in the community evenness between WM and BRD media. In contrast, YEM-grown communities had lower diversity and higher evenness, primarily explained by the reduction in the number and dominance of the most abundant members.

16S rRNA gene-based community composition profiling indicated that WM and BRD samples were abundant in the members of classes Clostridia and Bacteroidia (Figure 2C). Comparing WM and BRD-based community compositions, Erysipelotrichia and Clostridia were more abundant on a Western diet medium, whereas bread digest supported higher numbers of Negativicutes, Delta- and Gammaproteobacteria, and Synergistia. Some members of Bacilli fared well in the poor nutrient environment of the YEM medium.

**Figure 3:**
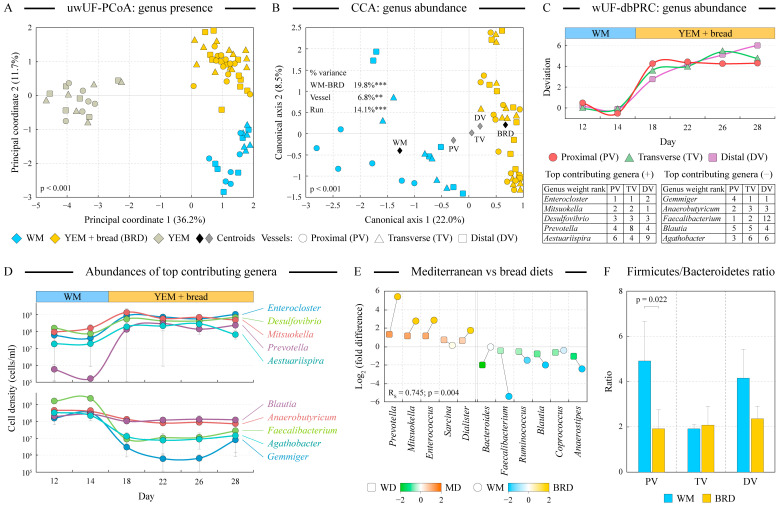
**Analysis of microbial community composition.** Panels (**A**,**B**) display the output of the unconstrained unweighted UniFrac distance-based principal coordinates analysis (UF-PCoA) and constrained canonical correspondence analysis (CCA) of the genus presence (panel (**A**)) and genus abundance (panel (**B**)) datasets. The percent of dataset variability explained by each axis is shown in parentheses in axis titles. Medium type, vessel identity, and replicate run were used as explanatory variables that constrained the variability of genus abundance dataset in the CCA analysis. For constraining categorical variables, position of each class centroid is indicated with a diamond. The analysis of variance of the CCA outputs revealed the relative contribution of explanatory variables to the overall variability in the dataset as shown; ***: *p* < 0.001, **: *p* < 0.01. Panel (**C**) displays the results of weighted UniFrac distance-based principal response curves (dbPRC) analysis of the genus abundance dataset. Consensus community composition on days 12 and 14 (representing stabilized community on Western diet medium) was set as the baseline for that vessel and was compared to the microbiota composition at every other time point. Larger deviation from zero on the Y axis represents a greater shift of community structure from that of the baseline. The main microbial drivers of these observed shifts are shown in the tables. Positive numbers represent genera that increased in their abundance after the medium switch; negative numbers represent genera that decreased. The changes in the average abundance of these genera at different time points are shown in panel (**D**). Note the logarithmic scale of Y axis. Error bars represent the standard error of the mean (*n* = 3). Panel (**E**) visualizes the comparison of the differences in the abundances of select genera between (i) distal gut microbiota communities of healthy adolescent subjects consuming either a Western diet (WD) or a Mediterranean diet (MD) (data taken from [43]) and between (ii) HGS distal vessel communities grown on WM and BRD media in this study. The ratios of the total Firmicutes to Bacteroidetes cell counts in each vessel are depicted in panel (**F**) as geometric means among all profiled samples of that phase. Statistical significance was assessed at the α = 0.05 level. Error bars represent the standard error of the mean (*n* = 3).

Both unconstrained (unweighted UniFrac distance PCoA) and constrained (CCA) genus level-based ordination analyses indicated that community structure differed among the three profiled medium compositions (*p* < 0.001, Figure 3A,B). Constrained CCA ordination analysis evaluated the contribution of several explanatory variables (medium type, vessel, and replicate run) to the overall variance of the microbial abundance dataset [39]. Comparing the communities of WM and BRD phases, the medium change was the largest contributor to the microbiota variability among samples, followed by replicate run and vessel identity (see Figure 3B). We utilized the weighted UniFrac distance-based principal response curves (dbPRC) [37] to track the changes in the genuscomposition of the communities over time. As visualized in Figure 3C, community composition was altered within four days after the switch from the WM to BRD medium in all three vessels, and dbPRC analysis identified the genera contributing the most to these alterations (see tables of contributing genera in Figure 3C panel). Temporal changes in the abundances of the top five increasing and decreasing genera are shown in Figure 3D. We found a noticeable concordance among the three vessels in the genera benefitting from the medium switch to the digested bread, with *Enterocloster*, *Mitsuokella*, and *Desulfovibrio* being the top three. *Enterocloster*, comprising recently reassigned *Clostridium* members, are gut commensals capable of producing microbial bile acids [40], but they also include opportunistic pathogenic members such as *E. bolteae*. *Mitsuokella* was observed to utilize lactate and produce propionate [41] and might thus be responsible for the increased propionate production observed during the BRD phase (see below). *Desulfovibrio* is a known sulfate-reducing bacterium whose expansion might be due to the high concentrations of sulfur-containing amino acids in flax seeds [42].

Genera reduced in abundance upon the switch to the BRD medium, included *Gemminger*, *Anaerobutyricum*, and *Faecalibacterium* (Figure 3D). These members are known butyrate-producing members in the gut microbiota, which is consistent with butyrate reduction observed during the BRD phase (see below).

### 3.4. Fermentation of Artisan Bread Promotes Community Changes Analogous to the Effect of Mediterranean Diet Consumption

We previously compared the composition of distal gut microbiota in healthy adolescent children consuming either a typical Western diet (WD) or a Mediterranean diet (MD) [43]. Mediterranean gut microbiota (assessed in the Egyptian population) was enriched in polysaccharide-degrading genome-encoded enzymatic functions, whereas WD microbiota (assessed in US subjects) had a higher frequency of protein-degrading and fat-utilization-encoded pathways and was also abundant in starch degraders. A number of genera differed significantly in their abundance between the WD and MD-consuming subjects, and we evaluated whether similar differences could be observed in the current study between WM and BRD-grown microbial communities. As visualized in Figure 3E, we revealed a strong, statistically significant concordance in the abundances of these genera among the two comparisons (Spearman correlation R_s_ = 0.745, *p* = 0.004). The genera plentiful in MD-consuming subjects (many degraders of complex polysaccharides, e.g., *Prevotella*, *Mitsuokella*) were also abundant in the BRD medium (in comparison with WM). The opposite was also true: genera dominant in the distal gut microbiota of WD-consuming subjects (these included *Faecalibacterium* and known starch degraders *Ruminococcus*, *Blautia*, and *Coprococcus*) were also more abundant in the WM medium. One notable exception was *Bacteroides*, which is significantly more abundant in the Western diet-consuming populations [44] but which displayed stable abundance between WM and BRD media in our experiments.

### 3.5. Firmicutes-to-Bacteroidetes Ratio Is Decreased upon the Switch from the Western to Digested Bread Medium

Because the Mediterranean diet has been associated with improved metabolic health [45], and because the changes in the Firmicutes-to-Bacteroidetes (FB) ratio were previously shown to be associated with obesity [46], we next examined the stability of the FB ratio during our HGS experiments in all three vessels. As shown in Figure 3F, the FB ratio was noticeably higher on WM than on BRD medium in the proximal (*p* = 0.022) and distal vessels (*p* > 0.05); no difference was evident in the transverse vessel.

**Figure 4:**
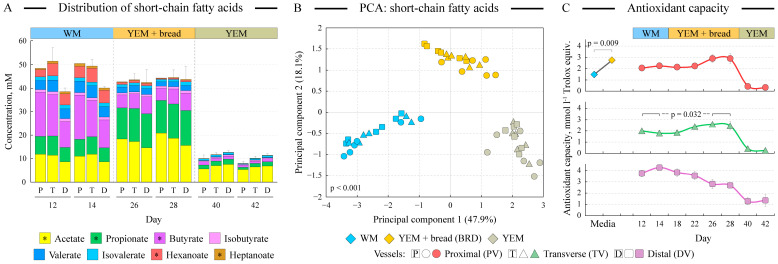
**Metabolite and antioxidant measurements.** Panel (**A**) visualizes the concentrations of measured short-chain fatty acids in all samples. Each column represents an average concentration among the three replicate runs; error bars represent the standard error of the mean of the total SCFA amount (*n* = 3). Stars in the legend denote statistical significance of abundance differences between WM and YEM + bread samples at the α = 0.05 level. PCA ordination analysis of the SCFA concentration dataset is shown in panel (**B**). The percent of dataset variability explained by each axis is shown in parentheses in axis titles. Panel (**C**) displays the antioxidant capacities of the freshly prepared WM and YEM + bread media and also shows the change in the average antioxidant capacity of cultures over time. Error bars represent the standard error of the mean (*n* = 3).

### 3.6. Composition of Fermentation End Products Differs between WM and BRD Media

We determined the concentrations of eight different short-chain fatty acids, the end products of microbial anaerobic fermentation, in all samples. While the total amount of produced SCFAs did not differ significantly between the WM and BRD-grown cultures, there was a noticeable difference in the composition of SCFAs (Figure 4A). Fermentation of the Western diet medium led to the accumulation of butyrate, followed by acetate and propionate. Fermentation of the digested UF bread, however, favored the production of acetate and propionate, consistent with the higher abundance of acetate and propionate producers *Enterocloster* and *Mitsuokella* in BRD cultures [47]. The reduction in butyrate production is concordant with the observed decreases in the abundances of prominent butyrate producers, including *Faecalibacterium*, *Roseburia*, *Blautia*, and *Anaerobutiricum,* in cultures grown on the BRD medium. The production of hexanoic and heptanoic acids was also diminished. YEM-grown cultures expectedly produced significantly lower amounts of SCFAs, with acetate contributing above 60% of that total (Figure 4A). All these differences were sufficient to separate WM, BRD, and YEM samples in the PCA ordination space with statistical significance (Figure 4B). Co-inertia analysis was performed to compare the distribution of samples in the SCFA PCA and genus-based PCoA (shown in Figure 3A) spaces. It revealed a statistically significant concordance between the two ordinations (R_v_ = 0.680, *p* < 0.001), indicating that SCFA production is linked to the composition of microbial communities in the profiled samples.

### 3.7. Antioxidant Capacity of Artisan Bread-Based Medium Is Higher Than That of WM

ABTS assay was used to measure the antioxidant capacities of fresh WM and BRD media and the amount of antioxidants in the collected samples. Freshly made BRD medium contained statistically significantly higher amounts of radical scavengers in comparison with fresh WM, as shown in Figure 4C (*p* = 0.009). This was translated to higher antioxidant capacities of microbial cultures of the proximal and transverse vessels (*p* = 0.098 and 0.032, respectively). This trend seemed to be reversed in the distal vessel (*p* = 0.055). In all three vessels, YEM-grown cultures had vastly lower antioxidant capacities, as expected.

**Figure 5:**
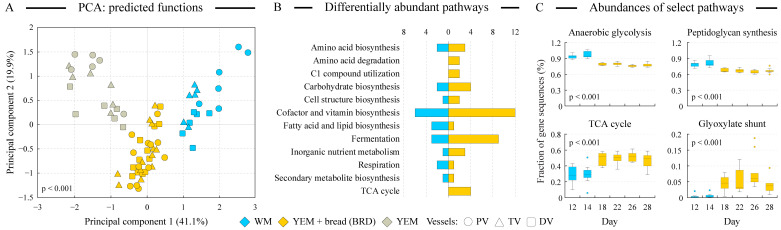
**Analysis of predicted functional capacities of microbial communities.** Panel (**A**) visualizes the distribution of samples in the PCA ordination space based on the frequencies of predicted metagenome-encoded microbial functions. The percent of dataset variability explained by each axis is shown in parentheses in axis titles. Panel (**B**) displays the numbers of statistically differentially encoded (DE) pathways in the predicted metagenomes of the WM and YEM + bread-maintained communities. Predicted functional genes were assigned to pathways and pathway groups according to the MetaCyc database [48]. Only pathway groups with at least two DE pathways are shown. The relative prevalence of several such pathways among WM and YEM + bread samples is shown in panel (**C**) as box-and-whisker plots. *p*-values represent the statistical significance of the difference in pathway frequency between WM and YEM + bread sample sets.

### 3.8. Functional Distinctions Separate WM and BRD Communities

By using the PICRUSt2 algorithm, we estimated the functional repertoire of community genomes. These repertoires were sufficiently distinct to separate the WM, BRD, and YEM sample groups in the PCA ordination space with statistical significance (Figure 5A), and a number of differentially encoded pathways were revealed (Figure 5B). The relative frequencies of several prominent pathways that differed between WM and BRD samples are displayed in Figure 5C. Overall, the bread-based medium supported fermentation pathways and the flux through the TCA and glyoxylate cycles. The former finding is likely because of the higher fiber content in the bread digest medium, and the latter observation is possibly due to the higher amount of total fats in the BRD medium providing higher availability of acetate. This last notion is consistent with the larger representation of the fatty acid biosynthesis pathways observed in WM-grown rather than BRD-grown cultures. Inorganic metabolism genes were encoded more frequently in the BRD cultures, consistent with the increase in *Desulfovibrio* abundance in bread digest samples and likely associated with the higher sulfur content of flax seeds [42]. The anaerobic glycolysis pathway was more prevalent among WM-supported microbes, potentially due to the substantial amount of starch and simple sugars present in the WM medium.

## 4. Discussion

We chose to investigate gut microbiota fermentation of UF bread because (i) it included no sources of gluten-containing grains, and (ii) it was loaded with dietary fiber and unsaturated fatty acids. The macronutrient composition of UF bread was listed (percent by weight) as 39% total carbohydrate, 21% protein, and 39% total fat, which compared favorably with those values for the standard white and wheat breads (83% carbs, 11% protein, and 6% fat, see Table 3). Macronutrient composition of the Western pattern diet is usually listed (percent by weight) as 60–62% carbohydrates, 18–20% proteins, and 18–20% fats [49]. The three main ingredients of the UF bread were almond flour, psyllium husks, and flax seeds, which explains the high dietary fiber and unsaturated fatty acid content (32% and 36% total macronutrients by weight, respectively) of this artisan bread formulation. Unsurprisingly, a significantly lower fraction of UF bread was digested by human OGI enzymes (29.6% by dry weight) than that for the typical supermarket white and wheat breads (76.6–79.4%), thus providing, in comparison, three times more nutrients to the resident colonic microbiota (see Table 1).

**Table 3:** Macronutrient composition of breads and diets, % weight.

*Macronutrient*	UF Bread *	White/Wheat Bread *	*Western Diet ^†^*	*Mediterranean Diet ^‡^*
Total carbs	39.3%	83.3%	61%	62%
Dietary fiber	32.1%	5.6%	7–10%	15%
Sugars	<3.6%	11.1%	18–22%	NG
Total protein	21.4%	11.1%	20%	21%
Total fats	39.3%	5.6%	19%	17%
Saturated fats	3.6%	<3.6%	9%	4%
Unsaturated fats	35.7%	<3.6%	10%	13%

* Data taken from product labels. ^†^ Data taken from [49] and the USDA Nutrient Content of the U.S. Food Supply 1909–2010 tables. ^‡^ Data taken from [50]; NG—value not given.

The digested UF bread was used to formulate a nutritional mix that was expected to reach the colon in a person after the consumption of UF bread. Such a BRD medium had significantly higher antioxidant contents compared with the Western diet-based medium, and this higher antioxidant capacity was retained throughout BRD medium fermentation by gut microbiota (see Figure 4C). UF bread digest supported dense and diverse human gut microbial communities. In comparison, the community density on the YEM control medium, which contained all of the same compounds as the BRD medium, with the exception of digested bread, was only about 1/10th that of the WM and BRD cultures (see Figure 2A).

Numerous alterations were revealed in the community structure and functions upon the medium switch from the Western diet-based to the bread digest-based medium, as follows:

- Bread digest supported a more diverse microbial community in the proximal vessel.
- Community structure alterations included increases in fiber degraders such as *Prevotella* and *Mitsuokella* on the BRD medium but also increases in potentially detrimental *Enterocloster*, *Desulfovibrio*, *Bilophila*, and *Escherichia*/*Shigella* as well as a reduction of total *Faecalibacterium*, the genus previously shown to possess potent anti-inflammatory properties [51]. Substantially higher fiber content of the BRD medium not only supported fiber degraders listed above but also increased the prevalence of encoded fermentation pathways in the community.
- The Firmicutes-to-Bacteroidetes ratio was reduced on the BRD medium in the proximal and distal vessels. A higher F-to-B ratio was previously found in some obese subjects [46]; thus, lowering this ratio on the bread digest medium might have beneficial effects on the host.
- Genomes of microbial communities grown on the bread digest medium were predicted to encode more functions in the pathways of the TCA cycle, glyoxylate bypass, and fatty acid degradation (see Figure 5B,C), all indicative of a higher acetyl-CoA utilization, consistent with the higher lipid content of the BRD medium. This was consistent with the higher abundance of *Bilophila*, a previously recognized “lipophilic” genus, in the BRD-grown cultures.
- Pathways of the inorganic nutrient metabolism were also more prevalent in the BRD-maintained communities, consistent with the increase of sulfate-reducing *Desulfovibrio* in BRD medium and high sulfur content of flax seeds.
- While the overall production of short-chain fatty acids was not statistically different between WM and BRD-grown communities, we observed a shift from the production of butyrate towards the release of propionate and acetate. This was in agreement with the reduction of the abundance of several butyrate producers (e.g., *Faecalibacterium*, *Blautia*, *Anaerobutiricum*) and an increase in propionate producers such as *Enterocloster* and *Mitsuokella* in the UF bread-grown communities. Both butyrate and propionate have been separately associated with protection from obesity and metabolic syndrome as well as eliciting a number of other health-associated benefits [50].

Our most intriguing finding was the discovered concordance of the alterations of gut microbiota composition between the Western diet and bread digest media and the differences in distal gut microbiota in subjects consuming Western vs. Mediterranean diets (see Figure 3E and reference [43]). In most cases, genera that were more abundant in the distal gut of Mediterranean-consuming subjects also increased in abundance after the medium was switched from WM to BRD in our HGS experiments. The opposite was likewise true—genera abundant in the Western diet were also more prevalent in WM-grown cultures. This is likely explained by the UF bread composition resembling the macronutrient content of a typical Mediterranean diet, with higher abundances of dietary fiber and unsaturated fatty acids and lower amounts of saturated fats (see Table 3 and references [44,52]). This finding provides additional credibility to the notion that diet composition has a stronger influence on microbiota structure in comparison with gut colonization history or the subject’s geographic location [53]. It also highlights that artisan breads, high in dietary fiber and unsaturated fat ingredients, might be able to at least partially recapitulate the beneficial effects of Mediterranean diets, even in industrialized populations.

Because our *in vitro* HGS system does not contain human components such as epithelial and immune cells and human tissue-derived molecules such as defensins and secretory IgAs, our investigations were limited to the elucidation of the direct effects of dietary components in the digested artisan bread on the human gut microbiota. Additional confounding factors are expected to influence the relationship between the diet and microbiota, including the indirect effects of nutrient components on human cells and tissues, in turn affecting the colonic environment and, thus, colonic microbes [54]. Based on our promising findings, further animal and clinical studies might be warranted to investigate the “holistic” effects of non-wheat-based breads on human physiology and gut health. These can also assess whether any expansion of potentially harmful microbiota members can have an effect on host health.

In summary, does the revealed evidence support the beneficial properties of the high fiber-based artisan breads? On the one hand, the higher fiber content and lower starch amounts in UF bread in comparison with wheat-based breads can reduce the risk of diabetes development. The lower F-to-B ratio of human gut microbiota grown on UF bread digest can also protect against obesity, and the higher antioxidant capacity of bread-based medium would have a protective effect against epithelial damage and might reduce the risk of gut diseases such as colorectal cancer [55]. On the other hand, bread digest supported an increased abundance of several detrimental microbial genera (*Desulfovibrio*, *Bilophila*, *Escherichia*/*Shigella*) and drastically reduced the numbers of beneficial *Faecalibacterium*. Finally, the shift of WM microbiota communities towards BRD-promoted expansion of members that are abundant in subjects consuming a Mediterranean diet seems to tip the scales towards an overall microbiota-beneficial label for the UF bread.

## Figures and Tables

**Table 1:** Digestion of breads and individual UF bread ingredients.

*Ingredient*	*Digested after OGI Process* *	*Solids Remaining after OGI Digestion* *
UF bread	29.6%	70.4%
Almond flour	65.2%	34.8%
Baking powder	71.4%	28.6%
Eggs	98.6%	1.4%
Flax seeds	23.3%	76.7%
Psyllium husks	<1%	99.0%
White bread	79.4%	20.6%
Whole wheat bread	76.6%	23.4%

* Shown values represent averages of two independent tests.

## Data Availability

The original contributions presented in the study are included in the article and supplementary table, further inquiries can be directed to the corresponding author.

## Supplementary Materials

The following supporting information can be downloaded at: https://www.mdpi.com/article/10.3390/microorganisms12061189/s1, Supplementary Table S1: cumulative genus-level cell densities in all samples.
