# Supplementary material for: Gut Microbiota Fermentation of Digested Almond–Psyllium–Flax Seed-Based Artisan Bread Promotes Mediterranean Diet-Resembling Microbial Community
Source: Microorganisms. 2024 Jun 12;12(6):1189. doi: 10.3390/microorganisms12061189 (PMC11205402; doi:10.3390/microorganisms12061189)
Supplement: Supplementary file 1 [file microorganisms-3037022-supplementary.pdf › microorganisms-3037022-supplementary.pdf]

Cell densities (x E06)	Run1.PV.D12	Run1.PV.D14	Run1.PV.D18	Run1.PV.D22	Run1.PV.D26	Run1.PV.D28	Run1.PV.D40	Run1.PV.D42	Run1.TV.D12	Run1.TV.D14	Run1.TV.D18	Run1.TV.D22	Run1.TV.D26	Run1.TV.D28	Run1.TV.D40	Run1.TV.D42	Run1.DV.D12	Run1.DV.D14	Run1.DV.D18	Run1.DV.D22	Run1.DV.D26	Run1.DV.D28	Run1.DV.D40	Run1.DV.D42	Run2.PV.D12	Run2.PV.D14	Run2.PV.D18	Run2.PV.D22	Run2.PV.D26	Run2.PV.D28	Run2.PV.D40	Run2.PV.D42	Run2.TV.D12	Run2.TV.D14	Run2.TV.D18	Run2.TV.D22
Acetatifactor	0	0	0	0	0	0	0	0	0	0	0	0	0	0	0	0	0	0	0	0	0	0	0	0	0	0	0	0	0	0	0	0	0	0	0	0
Acetobacteroides	0	0	0	0	0	0	0	0	0	0	0	0	0	0	0	0	0	0	0	0	0	0	0	0	0	1	0	0	0	0	0	0	0	0	0	0
Achromobacter	0	0	0	0	0	0	0	0	0	0	0	0	0	0	0	0	0	0	0	1	0	0	0	0	0	0	0	0	0	0	0	0	0	0	0	0
Acidaminococcus	0	0	0	0	0	0	0	0	0	0	0	0	0	0	0	0	0	0	0	0	0	0	0	0	0	0	0	0	0	0	0	0	0	0	0	0
Acinetobacter	0	0	0	0	0	0	0	0	0	0	0	0	0	0	0	0	0	0	0	0	0	0	0	0	0	0	0	1	0	0	0	0	0	0	0	0
Acutalibacter	14	7	0	0	0	0	0	0	1	6	0	0	0	0	0	0	1	3	0	0	0	0	0	0	7	6	0	0	0	0	0	0	3	1	0	0
Adlercreutzia	7	6	13	27	17	21	0	0	4	9	1	13	6	9	0	0	11	4	4	3	7	4	0	0	1	1	16	30	6	7	0	0	4	1	11	24
Aestuariispira	55	55	140	13	54	58	1	0	7	13	75	13	18	4	0	0	9	4	43	4	10	7	0	0	0	0	234	291	221	23	0	0	7	11	116	277
Agathobacter	31	34	27	4	14	3	0	0	26	21	14	3	4	4	0	1	10	9	7	4	1	4	0	0	284	217	1	10	6	7	0	1	183	126	3	7
Agathobaculum	7	3	17	17	75	111	0	1	9	6	11	11	33	37	0	0	4	0	10	9	11	6	1	0	0	0	34	37	35	10	1	0	1	1	18	26
Akkermansia	0	0	0	0	0	0	0	0	17	10	17	92	13	7	0	0	65	17	9	125	17	17	0	0	0	0	0	0	0	7	0	0	0	0	0	20
Alistipes	9	7	28	3	7	9	33	38	24	37	10	9	11	6	17	24	79	48	61	149	119	193	7	9	4	1	0	0	3	18	0	1	35	17	4	13
Allisonella	0	0	0	0	0	0	0	0	0	0	0	0	0	0	0	0	0	0	0	0	0	0	0	0	0	0	0	0	0	0	0	0	0	0	0	0
Aminipila	0	0	1	3	6	6	4	6	3	9	1	7	14	7	1	0	10	4	3	3	9	6	0	0	0	1	0	0	0	9	0	0	6	7	7	9
Amphibacillus	0	0	0	0	0	0	0	0	0	0	0	0	0	0	0	0	0	0	0	0	0	0	0	0	0	0	0	0	0	0	0	0	0	0	0	0
Anaerobacillus	0	0	0	0	0	0	0	0	0	0	0	0	1	0	0	0	0	0	0	0	0	0	0	0	0	0	1	0	1	1	0	0	0	0	1	0
Anaerobutyricum	437	535	92	122	108	92	0	1	448	387	150	119	152	169	0	0	200	260	201	116	94	146	1	0	632	417	111	74	109	43	0	0	684	679	77	38
Anaerococcus	0	0	0	0	0	0	0	0	0	0	0	0	0	0	0	0	0	0	0	0	0	0	0	0	0	0	0	0	0	0	0	0	0	0	0	0
Anaerofustis	0	1	0	1	1	0	0	0	0	0	0	0	1	0	0	0	0	0	0	0	0	0	0	0	0	0	0	0	0	0	0	0	0	0	0	0
Anaeromassilibacillus	1	1	0	0	0	0	0	0	0	1	0	0	0	0	0	1	4	1	0	0	0	0	1	0	3	1	0	0	0	0	0	0	9	1	0	0
Anaerosalibacter	0	0	0	0	0	0	0	0	0	0	0	0	0	0	0	0	0	0	0	0	0	0	0	0	0	0	0	0	0	1042	0	0	0	0	0	0
Anaerostipes	35	78	1	0	3	4	0	0	55	48	6	1	1	0	0	0	23	24	11	1	0	0	0	0	6	9	3	3	1	1	0	0	13	9	0	1
Anaerotignum	82	78	157	187	207	204	3	0	58	62	40	34	47	54	10	0	47	35	18	11	18	18	11	0	31	20	89	112	162	7	14	0	47	58	24	45
Anaerotruncus	14	9	6	3	4	4	7	17	64	44	6	4	7	9	7	16	89	69	7	6	4	10	13	16	0	0	0	0	3	1	1	0	9	9	7	0
Atlantibacter	0	0	0	0	0	0	0	0	0	0	1	0	0	0	0	0	0	0	0	0	0	0	0	0	0	0	0	0	0	0	0	0	0	0	0	0
Azotobacter	0	0	0	0	0	0	0	0	0	0	0	0	0	0	0	0	0	0	0	0	0	0	0	0	0	0	0	0	0	0	0	0	0	0	0	0
Bacillus	0	0	1	0	0	1	0	0	0	0	0	0	0	0	0	0	0	0	0	0	0	0	0	0	0	0	0	0	0	0	1	0	0	0	0	0
Bacteroides	1838	1113	850	862	954	906	55	62	1797	1768	580	732	789	644	55	45	1237	1488	743	611	757	688	34	37	1245	461	1614	1696	794	140	81	94	1821	1702	1526	1191
Bariatricus	0	1	1	3	1	3	0	0	4	3	1	3	0	1	1	0	0	3	0	0	0	1	1	0	1	1	6	4	1	1	0	0	0	0	0	3
Barnesiella	0	0	4	28	16	27	0	0	1	0	0	4	11	6	0	0	0	0	0	1	3	3	0	0	0	0	11	11	16	0	0	0	0	4	10	17
Beduinibacterium	0	0	0	0	0	0	0	0	1	1	0	0	0	0	0	0	1	1	0	0	0	0	0	0	0	0	0	0	0	0	0	0	0	0	0	0
Bifidobacterium	0	0	0	0	1	3	0	0	0	0	1	0	0	0	0	0	0	0	0	0	0	0	0	0	0	1	0	0	0	0	0	0	6	0	0	0
Bilophila	3	0	140	113	77	150	9	21	11	14	169	187	142	152	17	17	17	11	150	186	159	142	4	9	0	0	35	44	78	125	4	1	13	6	62	116
Blautia	349	498	122	167	286	203	0	1	630	606	67	115	150	190	0	3	457	632	16	21	30	26	1	0	155	208	96	133	91	52	3	0	221	247	65	128
Bradyrhizobium	0	0	0	0	0	0	0	0	0	0	0	0	0	0	0	0	0	0	0	0	0	0	0	0	0	0	0	0	0	0	0	0	0	0	0	0
Butyricicoccus	3	0	6	4	6	7	0	0	1	3	6	9	4	6	0	0	0	1	6	6	1	3	0	0	0	0	6	7	7	0	0	0	0	0	0	9
Butyricimonas	3	0	3	4	1	3	1	1	6	1	1	3	4	6	0	1	4	4	4	9	0	3	1	0	0	0	1	3	3	1	1	0	9	6	3	14
Butyrivibrio	0	0	1	0	0	0	0	0	0	0	0	0	0	0	0	0	0	0	0	0	0	0	0	0	0	0	0	0	0	0	0	0	0	0	0	0
Caproicibacter	1	0	0	0	0	0	0	0	0	1	0	0	0	0	0	1	0	1	0	0	0	0	0	1	3	0	0	0	0	0	0	0	1	0	0	0
Carnobacterium	0	0	0	0	0	0	0	0	0	0	1	0	1	0	0	0	0	0	1	0	0	0	0	0	0	0	0	0	0	0	0	0	0	0	0	1
Catabacter	0	0	0	0	0	0	0	0	1	0	0	0	0	0	0	0	0	1	0	0	0	0	0	0	0	0	0	0	0	0	0	0	1	1	0	0
Cedecea	0	0	0	0	0	0	0	0	0	0	0	0	0	0	0	0	0	0	0	0	0	0	0	0	0	0	0	0	0	0	0	0	0	0	0	0
Christensenella	0	0	0	0	0	0	0	0	0	0	0	0	0	0	0	0	0	0	0	0	0	0	0	0	0	0	0	0	0	0	0	0	0	0	0	0
Citrobacter	0	0	0	1	1	1	0	0	0	0	7	1	1	1	0	1	0	0	0	3	3	3	0	0	0	0	0	0	1	1	0	0	0	0	0	0
Cloacibacillus	0	0	6	10	4	6	3	4	9	13	6	17	7	10	6	9	24	20	26	62	50	51	7	6	0	0	0	1	0	13	0	0	6	0	7	11
ClostridiumIV	0	1	0	0	0	0	0	0	0	1	0	0	0	0	0	0	0	0	0	0	0	0	0	0	3	0	0	0	0	0	0	0	6	1	0	0
ClostridiumSS	0	1	1	4	4	1	0	0	0	0	0	0	3	1	0	0	1	1	0	0	0	0	0	0	1	0	0	0	0	16	0	0	0	1	0	0
ClostridiumXlVa	101	102	159	206	179	169	17	20	132	101	197	143	143	187	6	18	111	108	170	133	84	108	6	7	69	82	139	152	102	163	13	30	112	157	72	138
ClostridiumXlVb	13	7	0	0	0	0	0	0	7	1	0	0	0	0	0	0	1	0	0	0	0	0	0	0	1	0	0	0	0	0	0	0	1	0	0	0
Colidextribacter	9	40	10	3	10	10	0	0	16	33	14	0	38	11	0	0	17	20	9	0	31	7	0	0	0	0	0	0	0	4	0	0	28	62	3	9
Collinsella	26	3	0	4	0	0	0	0	9	17	1	6	0	0	0	0	10	14	0	4	1	0	0	0	34	28	0	0	1	0	0	0	79	45	1	0
Comamonas	0	0	0	0	0	0	0	0	0	0	0	0	0	0	0	0	0	0	0	0	0	0	0	0	0	0	0	0	0	0	0	0	0	0	0	0
Coprobacter	1	1	11	4	3	4	0	0	3	1	3	1	1	1	0	0	1	0	1	0	0	0	0	0	0	0	0	0	0	0	0	0	3	0	0	0
Coprococcus	24	48	16	30	38	21	0	0	47	27	13	16	28	23	1	0	10	10	4	6	3	7	6	1	16	35	17	30	18	6	0	0	13	20	9	9
Corynebacterium	0	0	0	0	0	0	0	0	0	0	0	0	0	0	0	0	0	0	0	0	0	0	0	0	0	0	0	0	0	0	0	0	0	0	0	0
Cronobacter	0	0	0	3	1	0	0	0	0	0	1	0	0	0	0	0	0	0	0	0	0	1	0	0	0	0	0	0	0	0	0	0	0	0	0	0
Cryptanaerobacter	0	0	0	0	0	0	0	0	1	3	0	0	0	0	0	0	1	1	0	0	0	0	0	0	0	0	0	0	0	0	0	0	0	0	0	0
Cuneatibacter	0	0	0	0	0	0	0	0	0	0	0	0	0	0	0	0	0	0	0	0	0	0	0	0	0	0	0	0	0	0	0	0	0	0	0	0
Cutibacterium	0	0	0	0	0	0	0	0	0	0	0	0	0	0	0	0	0	0	0	0	0	0	0	0	0	0	0	0	0	0	0	0	0	0	0	0
Desulfovibrio	116	94	1024	672	484	642	33	75	136	159	1122	1105	677	787	61	88	255	193	893	1271	672	753	59	47	164	61	204	316	474	674	18	11	425	187	306	549
Dialister	6	6	54	47	38	38	0	0	10	7	48	55	50	52	0	0	17	10	51	45	43	43	0	0	10	13	37	61	79	88	3	4	23	20	27	85
Dorea	7	9	9	33	67	31	0	0	6	3	11	31	84	62	0	0	1	6	4	38	47	50	0	0	4	35	17	17	13	28	0	0	13	11	24	17
Duncaniella	0	0	0	0	0	0	0	0	0	0	0	0	0	0	0	0	0	0	0	0	0	0	0	0	1	0	0	0	0	0	0	0	0	0	0	0
Duodenibacillus	0	0	17	104	31	48	0	0	0	0	14	65	13	20	0	0	1	0	7	14	9	4	0	0	72	7	102	213	145	14	7	10	142	10	55	98
Dysosmobacter	23	13	16	13	13	17	11	3	18	13	3	9	9	9	0	1	9	6	4	1	1	4	1	1	26	20	4	7	6	1	3	0	23	16	6	6
Eggerthella	0	0	0	0	0	1	0	0	0	0	0	0	0	1	0	0	0	1	0	0	0	0	0	0	0	0	0	0	0	0	1	0	1	0	0	0
Eisenbergiella	28	37	30	41	60	48	0	1	41	33	7	17	34	57	0	0	41	38	3	3	7	14	0	0	4	6	14	16	7	0	4	0	11	16	4	6
Enterobacter	0	0	0	0	0	1	0	0	1	0	3	0	1	0	0	0	0	0	0	0	1	1	0	0	0	0	0	0	0	0	0	0	0	0	0	0
Enterocloster	136	98	1302	1010	1152	772	18	52	421	224	1760	1020	1211	1517	11	60	593	403	1405	1024	1271	1268	11	20	51	20	1248	974	464	1641	31	52	267	298	1278	1020
Enterococcus	7	27	54	23	99	20	0	0	6	10	64	14	140	62	0	1	1	7	33	16	104	81	0	0	3	11	51	13	64	89	1	1	0	9	30	23
Erwinia	0	0	0	0	0	0	0	0	0	0	1	1	0	1	0	0	0	0	0	1	0	0	0	0	0	0	0	0	0	0	0	0	0	0	0	0
Escherichia/Shigella	11	40	176	262	518	201	4	20	4	37	125	214	475	191	4	7	4	16	37	147	353	224	52	43	9	4	11	10	136	58	23	4	3	20	13	18
Extibacter	0	0	0	0	0	0	0	0	1	0	1	0	0	3	0	0	1	1	0	0	0	0	0	0	0	0	0	0	0	0	0	0	0	0	0	0
Faecalibacillus	279	155	0	0	0	0	0	0	136	196	0	0	0	0	0	0	82	113	14	1	0	0	0	0	84	17	0	0	0	0	0	0	91	13	0	0
Faecalibacterium	1052	1157	0	0	1	3	0	0	96	122	1	3	1	0	0	0	4	13	0	0	0	0	0	0	2019	3302	6	1	3	0	10	7	340	648	0	3
Faecalimonas	9	10	6	7	26	18	0	0	11	9	0	4	11	17	0	0	11	4	1	1	1	1	0	0	1	1	0	0	1	0	0	0	1	0	0	0
Flavonifractor	7	7	0	0	3	0	10	11	7	4	0	0	0	0	4	17	4	7	0	0	0	0	11	10	3	14	0	0	0	0	54	10	3	11	0	0
Flintibacter	11	11	11	16	38	34	0	0	7	7	1	4	7	10	0	0	7	7	0	0	0	0	3	1	3	4	17	16	18	0	0	0	7	4	4	4
Fontibacillus	0	0	0	0	9	38	10	0	0	0	0	0	0	16	9	1	0	0	0	0	0	1	0	0	0	0	0	0	7	1	13	16	0	0	0	0
Fournierella	0	0	0	0	0	0	0	0	0	0	0	0	0	0	0	0	0	0	0	0	0	0	0	0	0	0	0	0	0	0	0	0	0	0	0	0
Fusicatenibacter	9	10	3	9	4	1	0	0	14	10	6	1	3	4	0	0	6	6	4	0	1	1	0	0	27	21	3	3	0	1	0	0	31	20	1	7
Gabonibacter	0	0	0	0	0	0	0	0	0	0	1	16	9	10	0	0	1	0	6	7	6	3	0	0	0	0	0	0	0	0	0	0	0	0	4	11
Gemmiger	60	318	0	1	0	1	0	0	44	301	7	0	0	0	0	1	20	27	17	0	0	0	0	0	67	52	1	0	0	0	0	0	123	65	4	0
Gibbsiella	0	0	0	0	0	0	0	0	0	0	0	0	0	0	0	0	0	0	0	0	0	0	0	0	0	0	0	0	0	0	0	0	0	0	0	0
Gracilibacillus	0	0	0	0	0	0	0	0	0	0	0	0	0	0	0	0	0	0	0	0	0	0	0	0	0	0	0	0	0	0	0	0	0	0	0	0
Granulicatella	1	0	1	0	3	0	0	0	0	1	1	1	4	0	0	0	0	0	1	0	3	3	0	0	0	0	1	1	1	1	0	0	0	0	1	3
Harryflintia	18	24	17	17	20	23	0	0	7	9	11	13	10	13	1	0	1	3	6	1	3	9	1	0	1	6	9	6	1	1	1	0	3	3	4	6
Herminiimonas	0	0	0	0	0	0	0	0	0	0	0	0	0	0	0	0	0	0	0	1	0	0	0	0	0	0	0	0	0	0	0	0	0	0	0	0
Hespellia	6	4	10	9	1	6	0	0	6	4	3	10	13	6	0	0	1	1	3	0	9	1	0	0	0	0	0	6	4	4	0	0	1	0	0	4
Hungatella	30	38	132	79	92	60	1	8	41	34	78	75	85	87	1	3	28	30	34	33	38	33	3	4	10	13	21	74	43	55	10	3	10	23	14	62
Hydrogeniiclostridium	0	0	0	0	0	1	0	0	0	0	0	0	0	0	0	0	0	0	0	0	1	0	0	0	0	0	0	0	0	0	0	0	0	1	0	0
Hydrogenoanaerobacterium	0	0	0	0	0	0	0	0	0	0	0	0	0	0	0	0	0	0	0	0	0	0	0	0	0	0	0	0	0	0	0	0	0	0	0	0
Ideonella	0	0	0	0	0	0	0	0	0	0	0	0	0	0	0	0	0	0	0	1	0	0	0	0	0	0	0	0	0	0	0	0	0	0	0	0
Ihubacter	4	3	17	16	6	3	0	3	13	9	17	24	10	6	3	4	31	24	18	11	10	9	3	1	1	1	3	3	0	14	0	0	13	11	9	9
Intestinibacillus	4	3	16	20	18	4	0	3	6	1	20	24	17	14	0	0	4	0	10	4	7	1	0	0	0	1	3	1	3	1	1	1	1	0	0	4
Intestinibacter	1	1	0	0	0	0	0	0	0	3	0	0	1	0	0	0	0	1	0	0	0	0	0	0	0	0	1	0	0	0	0	0	1	0	0	0
Intestinimonas	16	20	21	17	18	9	24	23	52	31	10	9	10	6	40	30	81	54	6	1	6	3	21	33	0	1	11	16	7	1	9	4	9	35	11	11
Isobaculum	0	0	3	0	3	0	0	1	0	0	1	1	3	0	0	0	0	0	1	0	3	4	0	0	0	0	4	0	0	4	0	0	0	0	1	0
Izhakiella	0	0	1	0	0	0	0	0	0	0	0	0	0	0	0	0	0	0	0	0	0	0	0	0	0	0	0	0	0	0	0	0	0	0	0	0
Kineothrix	0	1	1	0	0	1	0	0	0	1	1	0	1	1	0	0	0	0	1	0	1	1	0	0	0	0	0	0	0	0	0	0	0	0	0	1
Klebsiella	0	0	0	0	0	1	0	0	0	0	0	0	1	1	0	0	0	0	0	1	1	0	0	0	0	0	0	0	0	0	0	0	0	0	0	0
Kluyvera	0	0	1	1	0	0	0	0	0	0	1	0	1	0	0	0	0	0	0	1	0	1	0	0	0	0	0	0	0	0	0	0	0	0	0	0
Kocuria	0	0	0	0	0	0	0	0	0	0	0	0	0	0	0	0	0	0	0	0	0	0	0	0	0	0	0	0	0	0	0	0	0	0	0	0
Kosakonia	0	0	0	0	0	0	0	0	0	0	0	0	1	0	0	1	0	0	0	1	1	1	0	0	0	0	0	0	1	0	0	0	0	0	0	0
Lachnobacterium	0	0	0	4	1	0	0	0	0	0	0	0	0	0	0	0	0	0	0	0	0	0	0	0	0	0	0	1	3	0	0	0	0	0	0	0
Lachnospira	57	82	1	4	3	9	0	0	43	21	7	1	4	9	0	0	24	20	11	4	4	1	0	0	43	61	1	4	13	4	0	0	34	57	4	0

Cell densities (x E06)	Run2.TV.D26	Run2.TV.D28	Run2.TV.D40	Run2.TV.D42	Run2.DV.D12	Run2.DV.D14	Run2.DV.D18	Run2.DV.D22	Run2.DV.D26	Run2.DV.D28	Run2.DV.D40	Run2.DV.D42	Run3.PV.D12	Run3.PV.D14	Run3.PV.D18	Run3.PV.D22	Run3.PV.D26	Run3.PV.D28	Run3.PV.D40	Run3.PV.D42	Run3.TV.D12	Run3.TV.D14	Run3.TV.D18	Run3.TV.D22	Run3.TV.D26	Run3.TV.D28	Run3.TV.D40	Run3.TV.D42	Run3.DV.D12	Run3.DV.D14	Run3.DV.D18	Run3.DV.D22	Run3.DV.D26	Run3.DV.D28	Run3.DV.D40	Run3.DV.D42
Acetatifactor	0	0	0	0	0	0	0	0	0	0	0	0	0	0	0	0	0	1	0	0	0	0	0	0	0	0	0	0	0	0	0	0	0	0	0	0
Acetobacteroides	0	0	0	0	0	0	0	0	0	0	0	0	0	1	0	0	0	0	0	0	1	0	0	0	0	0	0	0	0	0	0	0	0	0	0	0
Achromobacter	0	0	0	0	0	0	0	0	0	0	0	0	0	0	0	0	0	0	0	0	0	0	0	0	0	0	0	0	0	0	0	0	0	0	0	0
Acidaminococcus	0	0	0	0	0	0	0	0	0	0	0	0	0	0	0	0	0	1	0	0	0	0	0	0	0	0	0	0	0	0	0	0	0	0	0	0
Acinetobacter	0	0	0	0	0	0	0	0	0	0	0	0	0	0	0	0	0	0	0	0	0	0	0	0	0	0	0	0	0	0	0	0	0	0	0	0
Acutalibacter	0	0	0	0	0	0	0	0	0	0	0	0	0	3	0	0	0	0	0	0	0	0	0	0	0	0	0	0	0	0	0	0	0	0	0	0
Adlercreutzia	20	11	0	0	1	1	35	14	6	16	1	0	3	6	0	1	0	6	0	0	3	3	1	0	1	0	0	0	1	1	0	0	1	1	0	0
Aestuariispira	148	4	0	4	0	1	4	45	31	88	0	0	0	1	216	411	597	102	0	7	4	10	166	82	620	657	0	1	0	0	61	41	129	85	0	0
Agathobacter	4	3	0	0	121	67	1	11	3	3	0	0	726	440	11	10	7	34	0	0	268	279	4	9	4	7	0	0	94	87	0	1	3	3	0	0
Agathobaculum	23	10	3	0	3	0	13	14	37	38	0	0	4	3	9	10	17	17	3	0	4	9	6	10	3	16	0	0	6	3	6	6	13	14	1	0
Akkermansia	10	4	0	0	0	0	0	33	4	0	0	0	0	0	0	0	0	33	0	0	0	0	0	0	0	1	1	0	0	0	0	4	3	7	0	0
Alistipes	7	21	0	1	6	7	4	4	0	1	6	0	16	7	1	1	1	13	4	1	23	9	6	9	3	3	0	0	10	23	4	88	79	136	0	0
Allisonella	0	1	0	0	0	0	1	0	0	0	0	0	0	0	0	0	0	0	0	0	0	0	0	0	0	0	0	0	0	0	0	0	0	0	0	0
Aminipila	10	20	1	3	11	11	4	17	4	0	0	0	0	0	0	0	0	6	0	0	7	9	1	0	0	3	0	1	6	10	1	0	1	16	0	0
Amphibacillus	0	0	0	0	0	0	0	0	0	0	0	0	0	0	0	0	0	0	0	0	0	0	0	0	0	0	0	0	9	10	0	0	0	0	0	0
Anaerobacillus	1	0	0	0	0	0	1	0	1	0	0	0	0	0	0	0	0	0	1	0	0	0	0	0	0	1	0	0	0	0	0	0	0	0	0	0
Anaerobutyricum	33	60	0	0	937	549	174	54	24	71	0	0	340	258	191	54	60	92	1	1	135	230	85	61	26	30	1	0	187	190	132	128	24	37	1	0
Anaerococcus	0	0	0	0	0	0	0	0	0	0	0	0	0	0	0	0	0	0	0	0	0	0	0	0	0	0	0	0	156	106	4	0	0	0	0	0
Anaerofustis	0	0	0	0	0	0	0	0	0	0	0	0	1	4	0	0	0	0	0	0	0	1	0	0	0	0	0	0	0	1	1	0	0	0	0	0
Anaeromassilibacillus	0	0	0	0	6	3	0	0	0	0	0	0	7	4	0	0	0	0	1	3	4	6	0	0	0	0	0	4	6	1	0	0	0	0	0	0
Anaerosalibacter	0	1543	0	0	647	675	709	1130	0	0	42	21	0	0	0	0	0	13	0	0	0	0	0	0	0	0	0	0	116	72	26	6	7	4	0	0
Anaerostipes	1	0	0	0	14	6	1	3	0	6	0	0	17	11	0	4	1	11	0	0	6	13	1	7	0	0	0	0	9	6	1	1	3	3	0	0
Anaerotignum	58	1	4	0	16	13	7	4	35	149	3	0	37	30	130	125	121	38	7	0	48	64	74	55	50	58	1	10	26	6	35	21	33	58	1	4
Anaerotruncus	4	3	1	0	0	1	0	1	1	3	0	0	1	0	1	0	0	7	0	0	0	4	0	3	0	1	0	1	1	1	0	4	7	21	1	0
Atlantibacter	0	0	0	1	0	0	0	0	0	0	0	0	0	0	0	0	0	0	0	0	0	0	0	0	0	0	0	0	0	0	0	0	0	0	0	0
Azotobacter	0	0	0	0	0	0	0	0	0	0	0	0	0	0	0	0	0	1	0	0	0	0	0	0	0	0	0	0	0	0	0	0	0	0	0	0
Bacillus	0	0	0	0	0	0	0	0	0	0	0	0	0	0	0	0	0	0	0	0	0	0	0	0	0	0	0	0	0	0	0	0	0	0	0	0
Bacteroides	705	113	70	74	316	491	355	251	424	498	61	77	227	152	922	2241	2285	1096	13	52	657	759	1149	2620	1665	1649	1	9	122	305	825	960	930	681	1	7
Bariatricus	0	0	0	0	1	0	0	0	0	3	0	0	0	1	3	0	0	0	0	0	1	1	6	0	0	4	0	1	0	1	0	3	0	3	1	0
Barnesiella	10	0	0	0	0	0	3	1	3	16	0	0	0	0	0	13	10	10	0	0	0	0	1	3	6	6	0	0	0	0	0	3	1	1	0	0
Beduinibacterium	0	0	0	0	0	1	0	0	0	0	0	0	0	0	0	0	0	0	0	0	0	0	0	0	0	0	0	0	0	0	0	0	0	0	0	0
Bifidobacterium	0	0	0	0	4	1	1	0	0	0	0	0	0	0	0	0	0	45	0	0	0	0	0	0	0	0	0	0	0	1	0	1	0	0	0	0
Bilophila	121	129	0	4	16	10	125	169	112	98	14	9	1	0	1	24	34	139	101	126	1	0	68	61	57	74	95	138	31	30	102	136	111	105	107	85
Blautia	78	34	0	1	79	57	20	62	58	67	0	1	95	139	132	68	38	106	0	0	88	149	115	142	31	47	0	1	101	85	92	88	44	89	0	0
Bradyrhizobium	0	0	0	0	0	0	0	0	0	0	0	0	0	0	0	0	0	3	0	0	0	0	0	0	0	0	0	0	0	0	0	0	0	0	0	0
Butyricicoccus	3	3	0	0	0	0	0	6	3	10	0	0	0	0	0	1	1	9	0	0	1	1	0	3	0	1	0	0	1	0	0	3	0	0	0	0
Butyricimonas	16	1	0	0	0	0	4	1	9	3	0	0	0	0	0	1	3	6	0	0	3	6	1	3	3	6	0	0	1	1	1	4	1	6	0	0
Butyrivibrio	0	0	0	0	0	0	0	0	0	0	0	0	0	1	0	0	0	0	0	0	0	0	0	0	0	1	0	0	0	0	0	0	0	0	0	0
Caproicibacter	0	0	0	0	0	0	0	0	0	0	0	0	0	1	0	0	0	0	0	0	0	0	0	0	0	0	0	0	0	0	0	0	0	0	0	0
Carnobacterium	1	1	0	0	0	0	1	0	0	0	0	0	0	0	0	0	0	0	0	0	0	0	0	0	0	0	0	0	0	0	0	0	0	0	0	0
Catabacter	0	0	0	0	0	0	0	0	0	0	1	0	0	0	0	0	0	0	0	0	0	0	0	0	0	0	0	0	0	0	0	0	0	0	0	0
Cedecea	0	0	0	0	0	0	0	0	0	0	0	0	0	0	0	0	0	1	0	0	0	0	0	0	0	0	0	0	0	0	0	0	0	0	0	0
Christensenella	0	0	0	0	0	0	0	0	0	0	0	0	0	0	0	0	0	0	0	0	0	0	0	0	0	0	0	0	0	0	0	0	0	0	1	0
Citrobacter	1	0	0	0	0	0	0	0	0	0	1	0	0	0	0	0	0	16	1	0	0	0	0	0	0	0	0	0	0	0	0	0	0	0	0	0
Cloacibacillus	6	28	0	11	4	3	21	28	4	0	8	0	0	0	0	0	0	17	4	10	0	0	0	1	1	3	6	21	0	0	3	4	3	10	16	21
ClostridiumIV	0	0	0	0	1	0	0	0	0	0	0	0	3	1	0	0	0	1	0	0	1	4	0	0	0	0	0	0	3	1	0	0	0	0	0	0
ClostridiumSS	0	26	0	0	9	17	6	33	0	0	0	0	0	0	0	1	0	9	0	0	0	0	0	0	0	0	0	0	0	0	0	0	1	1	0	0
ClostridiumXlVa	85	104	18	14	67	65	69	64	109	108	1	3	50	82	104	109	104	118	7	1	65	142	57	129	51	115	0	0	74	62	43	81	45	89	0	1
ClostridiumXlVb	0	0	0	0	0	0	0	0	0	0	0	0	1	1	0	0	0	0	0	0	0	3	0	1	0	3	0	0	0	0	0	0	1	3	0	0
Colidextribacter	3	4	0	0	34	64	4	4	4	0	0	0	0	0	0	0	0	1	0	0	26	3	1	0	0	0	0	0	16	3	3	3	0	3	0	0
Collinsella	0	1	0	0	62	64	18	0	0	0	0	0	78	14	4	0	3	10	0	0	34	23	3	0	1	0	0	0	18	23	11	4	0	0	0	0
Comamonas	0	0	0	0	0	0	0	0	0	0	0	0	0	0	0	0	0	1	0	0	0	0	0	0	0	0	0	0	0	0	0	0	0	0	0	0
Coprobacter	0	0	0	0	0	0	0	0	0	0	0	0	0	0	0	0	0	0	0	0	1	1	0	1	0	0	0	0	1	0	0	0	0	0	0	0
Coprococcus	11	7	0	1	11	10	9	4	26	16	1	0	7	13	31	1	3	9	10	0	4	11	18	1	0	3	7	1	4	3	3	0	0	6	3	1
Corynebacterium	0	0	0	0	0	0	0	0	0	0	0	0	0	0	0	0	0	1	0	0	0	0	0	0	0	0	0	0	0	0	0	0	0	0	0	0
Cronobacter	1	0	0	1	0	0	0	0	1	1	0	0	0	0	0	0	0	1	0	0	0	0	0	0	0	0	0	0	0	0	0	0	0	0	0	0
Cryptanaerobacter	0	1	0	0	0	0	0	0	0	0	0	0	0	0	0	0	0	0	0	0	0	0	0	0	0	0	0	0	0	0	0	0	0	0	0	0
Cuneatibacter	0	0	0	0	0	0	0	0	0	0	0	0	0	0	0	0	0	0	0	0	0	0	3	0	0	0	0	0	0	0	0	0	0	1	0	0
Cutibacterium	0	0	0	0	0	0	0	0	0	0	0	0	0	0	0	0	0	7	0	0	0	0	0	0	0	0	0	0	0	0	0	0	0	0	0	0
Desulfovibrio	522	720	58	68	294	245	340	440	611	458	54	91	217	72	481	312	330	661	64	65	349	224	241	346	363	311	120	112	275	336	451	730	352	328	55	80
Dialister	54	87	3	4	33	20	33	109	48	67	4	1	31	11	21	24	27	34	7	1	33	31	20	37	14	23	1	1	11	14	24	51	18	60	1	3
Dorea	13	37	0	0	11	6	26	9	9	13	1	0	136	210	7	7	13	44	0	0	45	68	9	13	13	17	0	0	6	10	6	6	9	38	1	0
Duncaniella	0	0	0	0	0	0	0	0	0	0	0	0	0	1	0	0	0	0	0	0	0	0	0	0	0	0	0	0	0	0	0	0	0	0	0	0
Duodenibacillus	62	21	7	18	11	3	24	31	85	145	1	4	0	0	6	4	4	35	0	0	0	1	6	7	3	3	0	0	0	0	1	3	1	4	0	0
Dysosmobacter	3	3	3	4	7	4	4	3	4	6	1	3	31	13	16	6	6	18	1	1	24	16	1	6	0	3	4	4	4	6	1	4	3	3	0	1
Eggerthella	1	0	1	0	0	0	0	0	0	0	0	0	0	0	0	0	0	11	0	0	0	0	0	0	0	0	0	0	0	0	0	0	0	0	0	0
Eisenbergiella	6	1	1	0	14	14	6	3	4	3	1	0	1	4	3	3	1	11	0	0	6	27	1	0	0	4	0	0	37	40	3	1	0	4	0	0
Enterobacter	0	0	0	0	0	0	0	0	0	0	0	0	0	0	0	0	0	1	0	0	0	0	0	0	0	0	0	0	0	0	0	0	0	0	0	0
Enterocloster	777	1013	31	38	309	352	1189	1227	400	260	27	31	13	7	71	113	116	579	0	6	74	89	204	200	142	221	1	11	172	102	262	299	201	379	3	9
Enterococcus	72	78	1	1	6	7	41	23	28	11	0	3	0	3	0	3	0	34	0	3	1	1	0	0	1	10	3	1	0	1	1	1	3	14	1	1
Erwinia	0	0	0	0	0	0	0	0	0	0	0	0	0	0	0	0	0	3	0	0	0	0	0	0	0	0	0	0	0	0	0	0	0	0	0	0
Escherichia/Shigella	169	74	18	17	3	7	4	7	142	82	8	9	1	0	0	0	0	389	55	34	1	0	0	3	0	0	64	21	1	0	1	1	1	7	136	71
Extibacter	0	0	0	0	0	0	0	0	0	0	0	0	0	0	0	0	0	0	0	0	0	0	0	0	0	0	0	0	0	0	0	0	0	0	0	0
Faecalibacillus	0	0	0	0	40	41	4	0	0	0	0	0	13	10	0	0	0	7	0	0	6	9	0	0	0	0	0	0	4	1	3	1	0	1	0	0
Faecalibacterium	1	0	6	3	122	87	3	0	1	1	0	3	1899	2798	21	30	30	87	0	0	374	555	3	6	4	0	0	0	16	30	3	1	1	3	0	0
Faecalimonas	0	1	0	0	0	0	0	0	1	0	0	0	3	0	0	0	1	3	0	0	0	1	4	0	0	1	0	0	4	3	1	0	0	0	0	0
Flavonifractor	0	0	43	13	3	6	0	0	1	0	7	1	1	4	0	0	0	16	4	9	4	7	0	0	0	0	1	1	0	1	1	0	0	0	1	1
Flintibacter	6	0	0	0	9	9	0	1	1	11	0	0	0	1	3	13	4	4	0	0	1	4	3	3	1	1	0	0	0	3	0	0	4	1	0	0
Fontibacillus	3	0	1	4	0	0	0	0	1	27	1	4	0	0	1	1	4	17	18	1	0	0	0	7	0	18	9	4	0	0	0	9	0	3	3	3
Fournierella	0	0	0	0	0	1	0	0	0	0	0	0	0	0	0	0	0	0	0	0	0	1	0	0	0	0	0	0	0	0	0	0	0	0	0	0
Fusicatenibacter	0	1	0	0	30	16	1	3	3	6	0	0	84	48	3	3	3	14	0	0	54	57	4	3	0	0	0	0	45	27	3	3	0	1	0	0
Gabonibacter	4	0	0	0	0	0	1	1	9	0	0	0	0	0	0	0	0	7	0	0	0	0	0	0	0	0	0	0	0	0	0	0	0	0	0	0
Gemmiger	1	1	0	0	193	145	4	1	0	1	0	0	406	603	7	0	1	24	0	0	1142	1051	30	1	0	0	0	0	757	1133	133	1	0	1	0	0
Gibbsiella	0	0	0	0	0	0	0	0	0	0	0	0	0	0	0	0	0	0	1	0	0	0	0	0	0	0	0	0	0	0	0	0	0	0	0	0
Gracilibacillus	0	0	0	0	0	0	0	0	0	0	0	0	0	0	0	0	0	0	0	0	0	0	0	0	0	0	0	0	3	1	0	0	0	0	0	0
Granulicatella	1	0	0	0	0	0	1	0	1	0	0	0	0	0	0	0	0	1	0	0	0	0	0	0	0	0	0	0	0	0	0	0	0	1	0	0
Harryflintia	7	1	1	0	0	3	6	0	3	6	0	0	10	11	7	4	7	3	4	0	4	11	9	6	9	7	0	0	4	1	0	4	6	17	0	0
Herminiimonas	0	0	0	0	0	0	0	0	0	0	0	0	0	0	0	0	0	0	0	0	0	0	0	0	0	0	0	0	0	0	0	0	0	0	0	0
Hespellia	0	4	0	0	1	0	1	1	0	1	0	1	1	0	0	1	0	9	0	0	0	0	1	0	0	0	0	0	0	1	1	0	0	0	0	0
Hungatella	41	40	6	4	9	11	11	26	44	48	3	1	7	10	14	18	17	41	30	17	26	44	28	27	13	18	6	6	23	20	14	14	11	38	1	1
Hydrogeniiclostridium	0	0	0	0	0	0	0	0	0	0	0	0	1	0	0	1	0	0	0	0	1	1	0	0	0	0	0	0	0	0	0	0	0	0	0	0
Hydrogenoanaerobacterium	0	0	0	0	1	0	0	0	0	0	0	0	0	0	0	0	0	0	0	0	0	0	0	0	0	0	0	0	0	0	0	0	0	0	0	0
Ideonella	0	0	0	0	0	0	0	0	0	0	0	0	0	0	0	0	0	0	0	0	0	0	0	0	0	0	0	0	0	0	0	0	0	0	0	0
Ihubacter	16	16	4	0	9	17	7	11	1	0	8	7	0	0	0	0	0	28	0	0	4	6	4	0	1	7	0	0	4	3	1	3	0	3	0	0
Intestinibacillus	1	3	0	0	0	0	1	0	3	3	0	0	0	1	0	3	1	13	0	0	1	3	1	6	3	1	0	1	1	3	0	1	1	7	0	0
Intestinibacter	0	0	0	0	1	0	0	0	0	0	0	0	3	4	1	0	0	3	0	0	6	6	0	0	0	0	0	0	3	4	3	0	0	0	0	0
Intestinimonas	4	1	20	9	27	31	6	0	6	7	4	3	3	4	3	0	1	30	1	0	38	41	1	3	1	6	0	1	30	30	4	4	3	7	1	3
Isobaculum	3	1	0	0	0	0	0	3	0	0	0	0	0	0	0	0	0	0	0	0	0	0	0	0	0	1	0	0	0	0	0	0	1	0	0	0
Izhakiella	0	0	0	0	0	0	0	0	0	0	0	0	0	0	0	0	0	0	0	0	0	0	0	0	0	0	0	0	0	0	0	0	0	0	0	0
Kineothrix	0	0	0	0	0	0	0	0	0	0	0	0	0	0	0	1	1	0	0	0	0	1	0	0	0	1	0	0	0	1	1	1	0	0	0	0
Klebsiella	0	0	0	0	0	0	0	0	0	0	0	0	0	0	0	0	0	3	0	0	0	0	0	0	0	0	0	0	0	0	0	0	0	0	0	0
Kluyvera	0	1	0	0	0	0	0	0	1	0	0	0	0	0	0	0	0	1	0	0	0	0	0	0	0	0	0	0	0	0	0	0	0	0	0	0
Kocuria	0	0	0	0	0	0	0	0	0	0	0	0	0	0	0	0	0	1	0	0	0	0	0	0	0	0	0	0	0	0	0	0	0	0	0	0
Kosakonia	0	0	0	0	0	0	0	0	0	0	1	0	0	0	0	0	0	4	0	0	0	0	0	0	0	0	0	0	0	0	0	0	0	0	0	1
Lachnobacterium	0	0	0	0	0	0	0	0	0	0	0	0	0	0	0	0	3	1	0	0	0	0	0	0	4	1	0	0	0	0	0	0	0	0	0	0
Lachnospira	1	3	0	0	28	9	9	0	0	3	0	0	35	47	6	3	1	4	0	0	1	34	0	3	0	3	0	0	9	3	1	3	0	0	0	0

Cell densities (x E06)	Run1.PV.D12	Run1.PV.D14	Run1.PV.D18	Run1.PV.D22	Run1.PV.D26	Run1.PV.D28	Run1.PV.D40	Run1.PV.D42	Run1.TV.D12	Run1.TV.D14	Run1.TV.D18	Run1.TV.D22	Run1.TV.D26	Run1.TV.D28	Run1.TV.D40	Run1.TV.D42	Run1.DV.D12	Run1.DV.D14	Run1.DV.D18	Run1.DV.D22	Run1.DV.D26	Run1.DV.D28	Run1.DV.D40	Run1.DV.D42
LachnospiracealS	60	77	13	78	24	7	0	0	58	51	4	6	14	4	0	0	26	27	1	1	4	1	0	0
Lachnotalea	1	0	0	0	0	0	0	0	1	0	1	0	0	0	0	0	1	1	0	0	0	0	0	0
Lacrimispora	4	1	51	21	35	14	1	1	9	3	60	17	50	26	0	0	9	7	38	20	35	24	0	0
Lactonifactor	0	0	0	0	0	1	0	1	0	0	0	0	0	0	0	0	0	0	0	0	0	0	0	0
Latilactobacillus	0	0	0	0	0	0	0	0	0	0	1	0	0	0	0	0	0	0	0	0	0	0	0	0
Lawsonella	0	0	0	0	0	0	0	0	0	0	0	0	0	0	0	0	0	0	0	0	0	0	0	0
Lawsonibacter	0	1	0	1	0	0	0	0	0	1	0	0	0	0	0	0	1	0	0	0	0	0	0	0
Longibaculum	0	0	0	0	0	0	0	0	0	0	0	0	0	0	0	0	0	0	0	0	0	0	0	0
Longicatena	37	16	0	0	0	0	0	0	23	31	0	0	0	0	0	0	37	18	1	0	0	0	0	0
Mailhella	0	0	0	0	0	0	0	0	0	0	0	1	0	0	0	0	0	0	0	0	0	0	0	0
Marseilla	3	0	6	58	10	31	0	0	0	0	4	23	10	14	0	0	0	1	1	4	7	1	0	0
Massiliimalia	1	1	0	1	1	3	0	0	3	0	0	0	3	1	0	0	1	1	1	0	0	0	0	0
Mediterranea	7	3	3	1	0	3	0	0	3	6	0	1	1	1	0	0	4	3	1	3	0	3	0	0
Mediterraneibacter	13	18	21	20	23	14	1	0	21	17	23	17	20	17	0	0	24	24	1	9	10	7	0	0
Melissococcus	0	0	1	1	1	0	0	0	0	0	0	0	0	0	0	0	0	0	0	0	0	0	0	0
Merdimonas	0	0	1	0	1	0	0	0	0	1	3	0	0	1	0	0	0	0	0	0	0	0	0	0
Methanobrevibacter	24	17	6	0	1	0	0	0	31	48	10	4	1	0	0	0	69	35	13	6	0	0	0	0
Methylobacterium	0	0	0	0	0	0	0	0	0	0	0	0	0	0	0	0	0	0	0	0	0	0	0	0
Micrococcus	0	0	0	0	0	0	0	0	0	0	0	0	0	0	0	0	0	0	0	0	0	0	0	0
Mitsuokella	169	203	50	35	240	81	0	0	179	190	112	24	363	143	1	0	78	99	152	13	183	125	0	0
Monoglobus	6	7	0	4	13	0	0	0	6	7	0	1	11	1	0	0	6	1	0	0	1	1	0	0
Mordavella	0	0	0	0	0	0	0	0	0	0	0	0	0	0	0	0	0	0	0	0	0	0	0	0
Mucispirillum	0	0	0	0	0	0	0	0	0	0	0	0	0	0	0	0	0	0	0	0	0	0	0	0
Muribaculum	81	48	7	1	1	1	0	0	68	78	9	1	1	0	0	0	38	35	0	3	0	0	0	0
Murimonas	6	10	6	0	6	1	0	0	10	7	3	6	4	1	0	0	3	0	0	0	0	1	0	0
Negativibacillus	4	9	9	6	4	6	0	1	7	11	7	3	1	6	3	3	4	1	3	1	0	1	0	3
Negativicoccus	1	1	0	0	0	0	0	0	1	6	1	0	0	0	0	0	3	1	0	0	0	0	0	0
Neglecta	6	11	0	0	1	0	0	0	1	7	0	0	0	1	0	1	0	1	0	0	0	0	0	0
Odoribacter	0	0	0	4	1	3	0	0	0	0	0	4	3	0	0	0	0	0	0	1	0	0	0	0
Olsenella	0	0	0	0	3	1	0	0	0	0	1	0	3	1	0	0	0	0	1	7	0	1	0	0
Oscillibacter	7	1	0	1	1	1	4	3	11	9	0	3	0	3	0	1	4	3	1	1	0	0	3	3
Oxalicibacterium	0	0	0	0	0	0	0	0	0	0	0	0	0	0	0	0	0	0	0	0	1	0	0	0
Oxalobacter	0	0	0	0	0	0	0	0	0	0	0	0	0	0	0	0	0	0	10	27	11	17	0	0
Paenibacillus	11	0	0	0	126	533	186	21	0	1	0	0	37	225	115	34	0	0	0	4	4	43	28	3
Paludicola	0	0	0	0	0	0	0	0	0	0	0	0	0	0	0	0	0	0	0	0	0	0	0	1
Pantoea	0	0	0	0	1	0	0	1	0	0	0	0	0	0	0	0	0	0	0	0	0	0	0	0
Parabacteroides	67	20	163	167	84	118	6	8	75	38	113	170	99	121	7	1	96	37	77	115	91	116	11	3
Parasutterella	43	23	166	108	71	180	10	41	44	35	99	82	94	122	6	28	23	6	24	37	45	61	3	71
Pelagirhabdus	0	0	0	0	0	0	0	0	0	0	0	0	0	0	0	0	0	0	0	0	0	0	0	0
Peptacetobacter	0	0	1	0	0	0	0	0	0	0	0	1	0	0	0	0	0	0	0	0	0	0	0	0
Peptoniphilus	0	0	0	0	0	0	0	0	3	1	0	0	0	0	0	0	9	9	0	0	0	0	0	0
Peredibacter	0	0	0	0	0	0	0	0	0	0	0	0	0	0	0	0	0	0	0	0	0	0	0	0
Peribacillus	0	0	0	0	1	0	0	0	0	0	0	0	0	0	0	0	0	0	0	0	0	0	0	0
Phascolarctobacterium	35	20	85	77	68	82	0	0	10	14	57	104	92	82	0	3	3	1	37	24	40	44	1	0
Phocaeicola	218	155	267	255	187	204	1	4	284	277	132	207	138	94	7	4	267	320	122	121	98	109	7	0
Phocea	37	34	170	109	89	54	9	3	58	54	115	92	89	78	10	4	58	35	14	23	30	21	4	1
Phycicoccus	0	0	0	0	0	0	0	0	0	0	0	0	0	0	0	0	0	0	0	0	0	0	0	0
Pilibacter	0	0	1	0	0	0	0	0	0	0	0	0	0	0	0	0	0	0	0	0	0	0	0	0
Pluralibacter	0	0	1	0	0	0	0	0	0	0	0	0	0	0	0	0	0	0	0	0	0	1	0	0
Prevotella	1	0	416	886	208	525	0	0	1	1	140	627	122	108	0	0	10	1	47	31	14	14	0	0
Pseudescherichia	1	6	31	24	30	35	0	4	0	9	20	26	23	13	1	3	1	0	6	18	23	21	1	0
Pseudoclostridium	0	0	0	0	0	0	0	0	1	0	0	0	0	0	0	0	3	1	0	0	0	0	0	0
Pseudoflavonifractor	1	0	0	0	0	0	0	1	1	0	0	0	0	0	0	0	1	1	0	0	0	0	1	0
Pseudomethylobacillus	0	0	6	16	10	13	0	0	0	1	3	7	7	1	0	0	0	0	0	6	10	3	0	1
Pseudomonas	0	0	0	0	0	0	0	0	0	0	0	0	0	0	0	0	0	0	0	0	0	0	0	0
Ralstonia	0	0	0	0	0	0	0	0	0	0	0	0	0	0	0	0	0	0	0	0	0	0	0	0
Raoultella	0	0	0	0	0	0	0	0	0	0	0	0	0	1	0	0	0	0	0	0	0	0	0	0
Rarimicrobium	0	0	0	0	0	0	0	0	7	17	4	10	6	0	0	0	31	48	14	9	3	7	0	0
Robinsoniella	0	3	1	1	0	0	0	0	1	0	1	1	0	0	0	0	1	0	0	0	0	0	0	0
Romboutsia	1	0	1	3	0	1	0	0	0	0	0	1	0	1	0	0	0	0	0	1	0	0	0	0
Roseburia	30	37	27	6	13	11	0	0	24	10	10	6	9	4	0	0	7	6	3	7	1	0	0	0
Rosenbergiella	0	0	0	0	1	0	0	0	0	0	0	0	1	0	0	0	0	0	0	0	0	0	0	0
Ruminococcus	17	31	28	58	72	89	0	0	14	14	18	17	33	34	0	0	6	7	0	1	6	4	0	0
Ruminococcus2	23	30	0	1	3	1	0	0	84	41	0	1	0	0	0	0	79	101	0	0	0	0	0	0
Ruthenibacterium	0	0	0	0	0	0	0	0	0	0	0	0	0	0	0	0	1	0	0	0	0	0	0	0
Sarcina	1	4	4	1	4	6	0	0	1	3	3	4	4	7	0	0	1	0	0	0	3	1	0	0
Schnuerera	0	0	0	0	0	0	0	0	0	0	0	0	0	0	0	0	0	0	0	0	0	0	0	0
Selenomonas	0	0	0	0	0	0	0	0	0	0	0	0	0	0	0	0	0	0	0	0	1	0	0	0
Sellimonas	43	30	4	1	6	4	0	0	62	37	4	6	1	1	0	0	79	52	3	1	0	4	0	0
Senegalimassilia	0	0	1	0	0	0	0	0	0	0	0	0	0	0	0	0	0	0	1	0	0	0	0	0
Serratia	0	0	0	0	0	0	0	0	0	0	0	0	1	0	0	0	0	0	0	0	0	0	0	0
Slackia	0	0	0	1	0	0	0	0	0	0	0	0	0	0	0	0	0	0	0	0	0	0	0	0
Sphingobium	0	0	0	0	0	0	0	0	0	0	0	0	0	0	0	0	0	0	0	0	0	0	0	0
Sphingomonas	0	0	0	0	0	0	0	0	0	0	0	0	0	0	0	0	0	0	0	0	0	0	0	0
Sporobacter	1	0	0	0	0	0	0	0	0	0	0	0	0	0	0	0	0	0	0	0	0	0	0	0
Staphylococcus	0	0	0	0	0	0	0	0	0	0	0	0	0	0	0	0	0	0	0	0	0	0	0	0
Streptococcus	0	0	1	0	1	0	0	0	0	0	1	0	1	0	0	0	0	1	0	0	0	0	0	0
Streptophyta	0	0	0	0	0	0	0	0	0	0	0	0	0	0	0	0	0	0	0	0	0	0	0	0
Subdoligranulum	11	27	0	0	0	0	0	0	7	30	1	0	0	1	0	0	10	0	1	0	0	1	0	0
Syntrophococcus	0	0	0	0	0	0	0	0	0	0	0	0	0	0	0	0	0	0	0	0	0	0	0	0
Tatumella	0	0	0	0	0	0	0	0	0	0	0	1	0	1	0	1	0	0	0	0	0	0	0	1
Tepidibaculum	1	0	0	1	0	1	0	0	1	4	3	0	0	0	0	0	0	1	0	0	0	0	0	0
Terrisporobacter	0	0	0	1	0	0	0	0	0	0	0	0	0	0	0	0	0	0	0	0	0	0	0	0
Tissierella	0	0	0	0	0	0	0	0	0	0	0	0	0	0	0	0	11	4	21	40	43	33	0	3
Unassigned	1325	1671	621	794	841	872	48	33	1049	1186	462	546	610	784	55	48	672	688	299	292	417	369	38	14
Ureibacillus	0	0	0	0	0	0	0	0	0	0	0	0	0	0	0	0	0	0	0	0	0	0	0	0
Urmitella	0	0	0	0	0	0	0	0	0	0	0	0	0	0	0	0	0	0	0	1	1	1	0	0
Vagococcus	0	0	0	0	0	0	0	0	0	0	0	0	0	1	0	0	0	0	1	0	0	1	0	0
Vampirovibrio	1	0	3	7	0	0	0	0	0	0	3	3	0	0	0	0	0	0	0	1	0	0	0	0
Victivallis	0	0	0	0	0	0	0	0	28	44	0	0	0	0	0	0	16	14	0	0	0	0	0	0
Virgibacillus	0	0	0	0	0	0	0	0	0	0	0	0	0	0	0	0	0	0	0	0	0	0	0	0
Wandonia	0	0	0	0	0	0	0	0	0	0	0	0	0	0	0	0	0	0	0	0	0	0	0	0
Weissella	0	0	0	0	0	0	0	0	0	0	0	0	0	0	0	0	0	0	0	0	0	0	0	0
Yersinia	0	0	0	0	0	0	0	0	0	0	0	0	0	1	0	0	0	0	0	0	0	0	0	0

Cell densities (x E06)	Run2.PV.D12	Run2.PV.D14	Run2.PV.D18	Run2.PV.D22	Run2.PV.D26	Run2.PV.D28	Run2.PV.D40	Run2.PV.D42	Run2.TV.D12	Run2.TV.D14	Run2.TV.D18	Run2.TV.D22	Run2.TV.D26	Run2.TV.D28	Run2.TV.D40	Run2.TV.D42	Run2.DV.D12	Run2.DV.D14	Run2.DV.D18	Run2.DV.D22	Run2.DV.D26	Run2.DV.D28	Run2.DV.D40	Run2.DV.D42
LachnospiracealS	9	11	17	34	34	6	0	0	9	7	10	4	4	1	0	0	4	4	3	0	3	13	0	0
Lachnotalea	1	3	0	0	0	0	0	0	1	3	0	0	0	0	0	0	1	1	0	0	0	0	1	0
Lacrimispora	0	0	6	3	9	10	0	0	0	3	9	3	0	7	1	0	1	3	6	0	1	1	1	0
Lactonifactor	0	0	0	0	0	0	0	0	0	0	0	0	0	0	0	0	0	0	1	0	0	0	0	0
Latilactobacillus	0	0	0	0	0	0	0	0	0	0	0	1	0	0	0	0	0	0	1	0	0	0	0	0
Lawsonella	0	0	0	0	0	0	0	0	0	0	0	0	0	0	0	0	0	0	0	0	0	0	0	0
Lawsonibacter	0	0	0	0	0	0	1	0	1	0	0	1	1	0	0	0	1	4	1	0	0	0	0	0
Longibaculum	38	30	1	0	0	0	0	0	17	34	0	0	0	0	0	0	11	41	1	0	0	0	0	0
Longicatena	3	1	0	0	0	0	0	0	3	0	0	0	0	0	0	0	4	1	0	0	0	0	0	0
Mailhella	0	0	0	0	0	0	0	0	0	0	0	0	0	1	0	0	0	0	0	0	0	0	0	0
Marseilla	6	4	33	21	37	3	0	0	3	1	23	21	30	6	0	0	0	0	20	6	14	20	0	0
Massiliimalia	0	1	0	0	0	0	0	0	0	0	0	1	0	0	0	0	1	0	0	0	1	0	0	0
Mediterranea	6	3	7	4	4	0	0	0	1	1	3	1	1	0	1	0	0	0	3	1	3	3	1	0
Mediterraneibacter	4	7	7	14	13	9	1	0	10	18	6	10	13	7	1	0	7	16	3	6	10	10	0	0
Melissococcus	0	0	0	0	0	1	0	0	0	0	0	0	1	0	0	0	0	0	0	0	0	0	0	0
Merdimonas	1	1	0	0	0	0	0	0	0	1	0	0	0	0	0	0	1	0	0	0	0	0	0	0
Methanobrevibacter	3	0	0	0	0	9	0	0	44	26	7	4	1	13	0	0	62	95	18	23	9	0	0	0
Methylobacterium	0	0	0	0	0	0	0	0	0	0	0	0	0	0	0	0	0	0	0	0	0	0	0	0
Micrococcus	0	0	0	0	0	0	0	0	0	0	0	0	0	0	0	0	0	0	0	0	0	0	0	0
Mitsuokella	109	282	1232	669	589	1198	0	0	87	390	1136	684	735	618	0	0	50	213	1024	434	519	132	0	0
Monoglobus	0	0	0	0	0	0	0	0	0	0	0	0	0	0	0	0	0	0	0	0	0	0	0	0
Mordavella	0	0	0	0	0	0	0	0	0	0	0	0	0	0	0	0	0	0	0	0	0	0	0	0
Mucispirillum	0	0	0	0	0	0	0	0	0	0	0	0	0	0	0	0	0	0	0	0	0	0	0	0
Muribaculum	11	3	3	7	4	1	0	0	9	3	0	9	4	0	0	0	0	0	1	3	0	3	0	0
Murimonas	0	0	4	0	9	0	0	0	0	1	0	3	0	0	0	0	0	0	0	0	0	0	0	0
Negativibacillus	4	9	3	0	0	1	0	0	9	13	1	0	3	0	0	0	6	4	1	1	1	0	0	0
Negativicoccus	0	0	0	0	0	0	0	0	0	0	0	0	0	0	0	0	0	0	0	0	0	0	0	0
Neglecta	13	9	0	0	0	0	0	0	1	1	0	0	0	0	0	0	0	0	0	0	0	0	0	0
Odoribacter	0	0	0	0	0	0	0	0	1	0	0	0	1	0	0	0	0	0	0	0	1	0	0	0
Olsenella	1	0	1	3	3	1	0	0	1	0	1	1	1	0	0	0	1	0	3	3	0	0	0	0
Oscillibacter	13	7	3	11	9	0	1	1	20	7	1	10	6	1	0	3	14	9	0	10	3	4	6	0
Oxalicibacterium	0	0	0	0	0	0	0	0	0	0	0	0	0	0	0	0	0	0	0	0	0	0	0	0
Oxalobacter	0	0	0	0	0	0	0	0	0	0	0	0	0	0	0	0	0	0	0	0	0	0	0	0
Paenibacillus	0	1	0	1	123	13	209	285	0	0	0	0	34	21	70	41	0	0	0	0	91	260	16	27
Paludicola	0	0	0	0	0	0	0	0	0	0	0	0	0	0	0	0	0	0	0	0	0	0	0	0
Pantoea	0	0	0	0	0	0	0	0	0	0	0	0	0	0	1	0	0	0	0	0	0	0	0	0
Parabacteroides	20	6	45	30	33	6	4	1	18	14	60	43	48	7	4	7	3	9	10	9	38	60	1	9
Parasutterella	0	0	7	3	4	0	7	20	0	0	6	3	7	4	4	21	1	1	4	1	3	6	4	6
Pelagirhabdus	0	0	0	0	0	0	0	0	0	0	0	0	0	0	0	0	0	0	0	0	0	0	0	0
Peptacetobacter	0	0	0	0	0	0	0	0	0	0	0	0	0	0	0	0	0	0	0	0	0	0	0	0
Peptoniphilus	0	0	0	0	0	0	0	0	1	0	0	0	0	0	0	0	3	1	0	0	0	0	0	0
Peredibacter	0	0	0	0	0	0	0	0	0	0	0	0	0	0	0	0	0	0	0	0	0	0	0	0
Peribacillus	0	0	0	0	0	0	0	0	0	0	0	0	0	0	0	0	0	0	0	0	0	0	0	0
Phascolarctobacterium	17	14	20	37	54	33	0	3	20	16	18	24	40	44	0	1	7	3	33	24	45	89	0	0
Phocaeicola	301	187	98	444	1203	234	1	1	370	265	189	450	804	189	6	1	247	196	84	326	496	460	6	3
Phocea	1	1	26	17	13	9	0	0	11	10	21	17	7	4	4	0	11	9	6	6	1	1	0	0
Phycicoccus	0	0	0	0	0	0	0	0	0	0	0	0	0	0	0	0	0	0	0	0	0	0	0	0
Pilibacter	0	0	0	0	0	0	0	0	0	0	0	0	0	0	0	0	0	0	0	0	0	0	0	0
Pluralibacter	0	0	0	0	0	0	0	0	0	0	0	0	0	0	0	0	0	0	0	0	0	0	0	0
Prevotella	0	0	0	1	213	13	0	0	0	1	3	21	238	3	0	0	0	0	0	0	678	800	0	0
Pseudescherichia	1	3	3	3	23	7	7	3	1	4	0	3	18	9	3	6	0	1	1	4	16	16	4	4
Pseudoclostridium	0	0	0	0	0	0	0	0	3	3	0	0	0	0	0	0	3	1	0	0	0	0	0	0
Pseudoflavonifractor	0	0	0	0	0	0	1	0	3	1	0	0	0	0	4	1	0	1	0	0	0	0	3	0
Pseudomethylobacillus	0	0	1	1	11	1	0	1	0	1	0	3	7	4	1	1	0	0	0	1	7	10	0	1
Pseudomonas	0	0	0	0	0	0	0	0	0	0	0	0	0	0	0	0	0	0	0	0	0	0	0	0
Ralstonia	0	0	0	0	0	0	0	0	0	0	0	0	0	0	0	0	0	0	0	0	0	0	0	0
Raoultella	0	0	0	0	0	0	0	0	0	0	0	0	0	0	0	1	0	0	0	0	0	0	0	0
Rarimicrobium	0	0	6	26	47	115	0	1	24	23	13	71	79	228	6	3	35	48	23	135	65	101	7	3
Robinsoniella	0	3	0	0	0	0	0	0	0	0	0	0	0	0	0	0	0	0	0	0	0	3	0	0
Romboutsia	1	1	0	1	0	0	0	0	1	1	0	0	0	0	0	0	1	0	0	0	0	0	0	0
Roseburia	173	115	7	6	4	7	0	0	77	51	6	9	7	0	0	0	54	26	3	1	6	1	0	0
Rosenbergiella	0	0	0	0	1	0	0	0	0	0	0	0	0	0	0	0	0	0	0	0	0	0	0	0
Ruminococcus	43	4	34	21	65	10	0	0	41	14	10	14	24	10	0	0	47	13	0	1	21	43	0	0
Ruminococcus2	17	45	0	0	1	0	0	0	58	136	0	0	0	0	0	0	45	115	1	0	0	0	0	0
Ruthenibacterium	0	1	0	0	0	0	0	0	0	0	0	0	0	0	0	0	0	0	0	0	0	0	1	0
Sarcina	1	1	1	0	3	1	0	0	0	3	3	1	0	1	0	1	0	1	1	0	0	4	0	0
Schnuerera	0	0	0	0	0	0	0	0	0	0	0	0	0	0	0	0	0	0	0	0	0	0	0	0
Selenomonas	0	0	0	0	0	0	0	0	0	0	0	0	0	0	0	0	0	0	0	0	0	0	0	0
Sellimonas	0	1	3	7	3	3	0	0	3	16	7	3	1	1	0	0	13	14	1	7	3	0	0	1
Senegalimassilia	13	1	0	0	0	0	0	0	26	6	1	0	0	0	0	0	13	20	7	0	0	0	0	0
Serratia	0	0	0	0	0	0	0	0	0	0	0	0	0	0	0	0	0	0	0	0	0	0	0	0
Slackia	0	0	1	1	0	0	0	0	0	0	0	0	0	0	0	0	0	0	0	6	0	0	0	0
Sphingobium	0	0	0	0	0	0	0	0	0	0	0	0	0	0	0	0	0	0	0	0	0	0	0	0
Sphingomonas	0	0	0	0	0	0	0	0	0	0	0	0	0	0	0	0	0	0	0	0	0	0	0	0
Sporobacter	0	0	0	0	0	0	0	0	0	1	0	0	0	0	0	0	0	0	0	0	0	0	0	0
Staphylococcus	0	0	0	0	0	0	0	0	0	0	0	0	0	0	0	0	0	0	0	0	0	0	0	0
Streptococcus	0	0	0	0	0	0	0	0	0	0	0	0	0	1	0	0	0	0	1	0	1	0	0	0
Streptophyta	0	0	0	0	0	0	0	0	0	0	0	0	0	0	0	0	0	0	0	0	0	0	0	0
Subdoligranulum	33	34	0	0	0	0	0	0	52	55	1	0	0	0	0	0	91	135	4	0	1	0	0	0
Syntrophococcus	0	0	0	0	0	0	0	0	0	0	0	0	0	0	0	0	0	0	0	0	0	0	0	0
Tatumella	0	0	0	0	0	1	1	0	0	0	0	0	0	0	0	0	0	0	0	0	0	0	0	0
Tepidibaculum	0	0	0	0	0	0	0	0	0	0	0	0	0	0	0	0	0	0	0	0	0	0	0	0
Terrisporobacter	0	0	0	0	0	0	0	0	0	0	0	0	0	0	0	0	0	0	0	0	0	0	0	0
Tissierella	0	0	0	0	0	0	0	0	0	0	0	0	0	0	0	0	0	0	0	0	0	0	0	0
Unassigned	1143	1103	417	586	709	274	116	92	740	781	346	484	512	309	51	58	794	885	301	226	647	711	44	60
Ureibacillus	0	0	0	0	0	0	0	0	0	0	0	0	0	0	0	0	0	0	0	0	0	0	0	0
Urmitella	0	0	1	0	0	0	0	0	0	0	0	0	0	0	0	0	0	0	0	0	0	0	0	0
Vagococcus	0	0	0	0	1	0	0	0	0	0	0	0	1	1	0	0	0	0	0	1	1	0	0	0
Vampirovibrio	0	0	0	0	0	0	0	0	0	0	0	0	0	0	0	0	0	0	0	0	0	0	0	0
Victivallis	0	0	0	0	0	0	0	0	10	7	1	0	0	0	0	0	0	4	1	0	0	0	0	0
Virgibacillus	0	0	0	0	0	0	0	0	0	0	0	0	0	0	0	0	0	0	0	0	0	0	0	0
Wandonia	0	0	0	0	0	0	0	0	0	0	0	0	0	0	0	0	0	0	0	0	0	0	0	0
Weissella	0	0	0	0	0	0	0	0	0	0	0	0	0	0	0	0	0	0	0	0	0	0	0	0
Yersinia	0	0	0	0	0	0	0	0	0	0	0	0	0	0	0	0	0	0	0	0	0	0	0	0

Cell densities (x E06)	Run3.PV.D12	Run3.PV.D14	Run3.PV.D18	Run3.PV.D22	Run3.PV.D26	Run3.PV.D28	Run3.PV.D40	Run3.PV.D42	Run3.TV.D12	Run3.TV.D14	Run3.TV.D18	Run3.TV.D22	Run3.TV.D26	Run3.TV.D28	Run3.TV.D40	Run3.TV.D42	Run3.DV.D12	Run3.DV.D14	Run3.DV.D18	Run3.DV.D22	Run3.DV.D26	Run3.DV.D28	Run3.DV.D40	Run3.DV.D42
LachnospiracealS	4	14	0	48	45	14	0	0	4	11	10	18	14	9	0	0	4	1	0	7	10	23	0	0
Lachnotalea	1	1	0	0	0	0	0	0	0	1	0	0	0	1	0	0	0	1	0	0	0	0	0	0
Lacrimispora	0	0	0	1	0	7	0	0	0	1	1	0	0	0	0	0	0	0	4	1	1	1	0	0
Lactonifactor	0	0	0	0	0	0	0	0	0	0	0	0	0	0	0	0	0	0	0	0	0	0	0	0
Latilactobacillus	0	0	0	0	0	0	0	0	0	0	0	0	0	0	0	0	0	0	0	0	0	0	0	0
Lawsonella	0	0	0	0	0	27	0	0	0	0	0	0	0	0	0	0	0	0	0	0	0	0	0	0
Lawsonibacter	0	0	0	0	0	3	1	0	0	1	0	0	0	0	0	0	0	1	0	0	0	1	0	0
Longibaculum	45	18	0	0	0	1	0	0	24	9	0	0	0	0	0	0	1	7	0	0	0	0	0	0
Longicatena	6	6	0	0	0	1	0	0	11	9	0	0	0	0	0	0	16	9	0	0	0	0	0	0
Mailhella	0	0	0	0	0	0	0	0	0	0	0	0	0	0	0	0	0	0	0	0	0	0	0	0
Marseilla	7	4	7	38	33	35	0	0	3	4	9	33	14	40	0	0	0	0	6	13	14	31	0	0
Massiliimalia	0	0	0	0	0	1	0	0	0	0	0	1	0	0	0	0	1	0	0	0	0	1	0	0
Mediterranea	1	0	9	10	11	7	0	1	1	0	6	7	7	7	0	0	0	0	3	9	7	3	0	0
Mediterraneibacter	7	9	7	7	1	17	0	0	10	18	4	10	3	4	0	0	17	24	6	3	3	6	0	0
Melissococcus	0	0	0	0	0	0	0	0	0	0	0	0	0	0	0	0	0	0	0	0	0	0	0	0
Merdimonas	0	1	3	10	16	0	0	0	0	3	1	11	1	10	1	0	0	1	1	1	3	1	0	0
Methanobrevibacter	1	0	0	0	0	1	0	0	13	18	0	0	0	0	0	0	26	40	7	3	3	7	0	0
Methylobacterium	0	0	0	0	0	4	0	0	0	0	0	0	0	0	0	0	0	0	0	0	0	0	0	0
Micrococcus	0	0	0	0	0	1	0	0	0	0	0	0	0	0	0	0	0	0	0	0	0	0	0	0
Mitsuokella	6	4	2884	1099	1082	389	0	0	98	18	2577	695	1746	591	0	0	138	43	2059	991	2185	1231	1	0
Monoglobus	0	0	0	0	0	1	0	0	0	0	0	0	0	0	0	0	0	0	0	0	0	0	0	0
Mordavella	0	0	0	0	0	0	0	0	0	0	1	0	0	0	0	0	0	0	0	0	0	0	0	0
Mucispirillum	0	0	0	0	0	1	0	0	0	0	0	0	0	0	0	0	0	0	0	0	0	0	0	0
Muribaculum	4	1	0	1	1	1	0	0	20	6	1	0	1	1	0	0	3	0	0	1	3	3	0	0
Murimonas	1	0	1	0	1	9	0	0	1	0	1	1	1	0	0	0	0	0	0	1	3	0	0	0
Negativibacillus	3	3	0	4	0	1	0	0	3	9	4	1	0	1	1	0	7	7	1	1	3	4	0	0
Negativicoccus	0	0	0	0	0	0	0	0	0	0	0	0	0	0	0	0	0	0	0	0	0	0	0	0
Neglecta	0	1	0	0	0	0	0	0	0	0	0	0	0	0	0	0	0	0	0	0	0	0	0	0
Odoribacter	0	0	0	1	6	1	0	0	0	0	3	0	1	0	0	0	0	0	0	0	0	0	0	0
Olsenella	0	0	0	1	1	1	0	0	1	0	0	0	1	0	0	0	1	0	1	6	1	1	0	0
Oscillibacter	3	6	3	0	1	7	0	1	7	10	0	1	1	3	0	0	6	9	3	3	1	3	0	0
Oxalicibacterium	0	0	0	0	0	0	0	0	0	0	0	0	0	0	0	0	0	0	0	0	0	0	0	0
Oxalobacter	0	0	0	0	0	0	0	0	0	0	0	0	0	0	0	0	0	0	0	7	1	4	1	1
Paenibacillus	0	0	4	26	38	132	352	228	0	0	6	58	4	217	164	115	0	0	1	106	3	119	41	95
Paludicola	0	0	0	0	0	0	0	0	0	0	0	0	0	0	0	0	0	1	0	0	0	0	0	0
Pantoea	0	0	0	0	0	1	0	0	0	0	0	0	0	0	0	0	0	0	0	0	0	0	0	0
Parabacteroides	94	35	48	44	38	96	6	17	55	30	40	26	14	35	4	7	6	4	23	26	17	23	0	3
Parasutterella	0	0	26	85	79	77	0	0	13	3	26	113	44	61	0	0	0	0	14	78	41	58	0	0
Pelagirhabdus	0	0	0	0	0	0	0	0	0	0	0	0	0	0	0	0	1	0	0	0	0	0	0	0
Peptacetobacter	0	0	0	0	0	1	0	0	0	0	1	0	0	0	0	0	0	1	0	0	0	0	0	0
Peptoniphilus	0	0	0	0	0	0	0	0	0	0	0	0	0	0	0	0	1	0	0	0	0	0	0	0
Peredibacter	0	0	0	0	0	1	0	0	0	0	0	0	0	0	0	0	0	0	0	0	0	0	0	0
Peribacillus	0	0	0	0	0	0	0	0	0	0	0	0	0	0	0	0	0	0	0	0	0	0	0	0
Phascolarctobacterium	11	9	33	13	17	47	1	3	13	11	26	13	13	9	0	3	11	1	30	31	20	33	0	1
Phocaeicola	954	262	411	589	535	284	1	6	1299	671	347	305	299	437	1	1	262	339	182	478	356	451	0	0
Phocea	3	0	4	6	6	33	0	4	7	10	24	3	4	13	4	6	16	16	6	1	3	7	1	0
Phycicoccus	0	0	0	0	0	3	0	0	0	0	0	0	0	0	0	0	0	0	0	0	0	0	0	0
Pilibacter	0	0	0	0	0	0	0	0	0	0	0	0	0	0	0	0	0	0	0	0	0	0	0	0
Pluralibacter	0	0	0	0	0	1	0	0	0	0	0	0	0	0	0	1	0	0	0	0	0	0	0	0
Prevotella	0	0	0	0	0	271	0	0	0	1	0	0	0	0	0	0	0	0	1	0	0	4	0	0
Pseudescherichia	0	0	0	0	0	37	17	11	0	0	0	0	0	1	4	1	0	0	1	0	0	0	9	9
Pseudoclostridium	0	0	0	0	0	0	0	0	6	0	3	0	0	0	0	0	7	4	0	0	0	0	0	0
Pseudoflavonifractor	0	0	0	0	0	0	0	0	1	0	0	0	0	0	0	0	0	0	0	0	0	0	0	0
Pseudomethylobacillus	0	0	0	0	0	10	1	1	0	0	0	0	0	0	1	0	0	0	0	0	0	0	1	1
Pseudomonas	0	0	0	0	0	3	0	0	0	0	0	0	0	0	0	0	0	0	0	0	0	0	0	0
Ralstonia	0	0	0	0	0	3	0	0	0	0	0	0	0	0	0	0	0	0	0	0	0	0	0	0
Raoultella	0	0	0	0	0	1	0	0	0	0	0	0	0	0	0	0	0	0	0	0	0	0	1	0
Rarimicrobium	0	0	0	0	0	13	0	0	108	77	10	7	4	6	0	0	207	200	18	41	16	57	0	0
Robinsoniella	0	0	3	0	0	1	0	0	0	1	0	0	0	0	0	0	0	0	0	0	0	0	0	0
Romboutsia	1	1	1	1	1	6	0	0	1	0	0	1	0	0	0	0	0	0	0	0	0	0	0	0
Roseburia	604	533	3	4	11	27	0	0	116	233	3	16	6	7	0	0	47	33	3	6	7	11	0	0
Rosenbergiella	0	0	0	0	0	0	0	0	0	0	0	0	0	0	0	0	0	0	0	0	0	0	0	0
Ruminococcus	88	35	13	18	35	28	0	0	85	45	9	4	10	34	0	0	21	21	6	3	10	31	0	0
Ruminococcus2	18	61	1	3	0	4	0	0	24	81	1	0	0	0	0	0	54	60	0	0	1	0	0	0
Ruthenibacterium	0	0	0	0	0	0	0	0	0	0	0	0	0	0	0	0	0	0	0	0	0	0	0	0
Sarcina	1	0	0	1	1	3	0	0	3	0	1	1	1	0	0	0	0	3	1	1	0	1	0	0
Schnuerera	0	0	0	0	0	0	0	0	0	0	0	0	0	0	0	0	9	9	0	0	0	0	0	0
Selenomonas	0	0	0	0	0	0	0	0	0	0	0	0	0	0	0	0	0	0	0	0	0	0	0	0
Sellimonas	0	0	1	1	3	6	0	0	4	21	1	0	0	0	0	0	23	18	1	1	1	1	1	0
Senegalimassilia	6	3	1	6	3	6	0	0	27	3	3	3	6	4	0	0	10	11	1	0	4	16	0	0
Serratia	0	0	0	0	0	0	0	0	0	0	0	0	0	0	0	0	0	0	0	0	0	0	0	1
Slackia	0	0	0	0	0	0	0	0	0	0	0	0	0	0	0	0	1	1	0	0	0	0	0	0
Sphingobium	0	0	0	0	0	1	0	0	0	0	0	0	0	0	0	0	0	0	0	0	0	0	0	0
Sphingomonas	0	0	0	0	0	3	0	0	0	0	0	0	0	0	0	0	0	0	0	0	0	0	0	0
Sporobacter	0	0	0	0	0	3	0	0	0	0	0	0	0	0	0	0	0	0	0	0	0	0	0	0
Staphylococcus	0	0	0	0	0	4	0	0	0	0	0	0	0	0	0	0	0	0	0	0	0	0	0	0
Streptococcus	0	0	0	0	0	0	0	0	0	0	0	0	0	0	0	0	0	0	0	0	0	0	0	0
Streptophyta	0	0	0	0	0	3	0	0	0	0	0	0	0	0	0	0	0	0	0	0	0	0	0	0
Subdoligranulum	31	34	1	0	0	4	0	0	69	38	6	0	0	0	0	0	27	48	11	0	0	0	0	0
Syntrophococcus	0	0	0	0	0	0	0	0	0	0	0	0	0	0	0	0	1	1	0	0	0	0	0	0
Tatumella	0	0	0	0	0	1	0	0	0	0	0	0	0	0	0	0	0	0	0	0	0	0	0	0
Tepidibaculum	0	0	0	0	0	0	0	0	0	0	0	0	0	0	0	0	0	0	0	0	0	0	0	0
Terrisporobacter	0	0	0	0	0	0	0	0	0	0	0	0	0	0	0	0	0	0	0	0	0	0	0	0
Tissierella	0	0	0	0	0	1	0	0	0	0	0	0	0	0	0	0	75	81	26	0	3	4	0	0
Unassigned	584	854	719	912	860	810	78	74	587	864	485	912	508	912	44	21	643	515	252	396	262	562	38	28
Ureibacillus	0	0	0	0	0	0	0	0	0	0	0	0	0	0	0	0	0	0	0	0	0	0	0	1
Urmitella	0	0	0	0	0	4	0	0	0	0	0	1	0	0	0	0	1023	728	35	16	6	6	0	0
Vagococcus	0	0	0	1	0	0	0	0	0	0	0	0	0	0	0	0	0	0	0	0	0	0	0	0
Vampirovibrio	0	0	0	0	0	6	0	0	0	0	0	41	0	48	0	0	0	0	0	18	1	13	0	0
Victivallis	0	0	0	0	0	0	0	0	0	0	0	1	0	0	0	0	0	0	3	1	0	0	0	0
Virgibacillus	0	0	0	0	0	0	0	0	0	0	0	0	0	0	0	0	1	1	0	0	0	0	0	0
Wandonia	0	0	0	0	0	1	0	0	0	0	0	0	0	0	0	0	0	0	0	0	0	0	0	0
Weissella	0	0	0	0	0	1	0	0	0	0	0	0	0	0	0	0	0	0	0	0	0	0	0	0
Yersinia	0	0	0	0	0	1	0	0	0	0	0	0	0	0	0	0	0	0	0	0	0	0	0	0
